# Extracellular vesicles as a promising source of lipid biomarkers for breast cancer detection in blood plasma

**DOI:** 10.1002/jev2.12419

**Published:** 2024-03-05

**Authors:** Erika Dorado, Maria Luisa Doria, Anika Nagelkerke, James S. McKenzie, Stefania Maneta‐Stavrakaki, Thomas E. Whittaker, Jeremy K. Nicholson, Raoul Charles Coombes, Molly M. Stevens, Zoltan Takats

**Affiliations:** ^1^ Faculty of Medicine, Department of Metabolism, Digestion and Reproduction Imperial College London London United Kingdom; ^2^ Faculty of Engineering, Department of Bioengineering, Department of Materials, Institute of Biomedical Engineering Imperial College London London United Kingdom; ^3^ Institute of Global Health Innovation Imperial College London London United Kingdom; ^4^ Faculty of Medicine, Department of Surgery and Cancer Imperial College London London United Kingdom; ^5^ PRISM Inserm U1192 University of Lille Lille France; ^6^ Deparment of Immunomedicine University of Regensburg Regensburg Germany

**Keywords:** breast cancer, extracellular vesicles, lipidomics, liquid biopsy, mass spectrometry

## Abstract

Extracellular vesicles (EVs), including exosomes and microvesicles, mediate intercellular communication in cancer, from development to metastasis. EV‐based liquid biopsy is a promising strategy for cancer diagnosis as EVs can be found in cancer patients’ body fluids. In this study, the lipid composition of breast cancer‐derived EVs was studied as well as the potential of blood plasma EVs for the identification of lipid biomarkers for breast cancer detection. Initially, an untargeted lipidomic analysis was carried out for a panel of cancerous and non‐cancerous mammary epithelial cells and their secreted EVs. We found that breast cancer‐derived EVs are enriched in sphingolipids and glycerophospholipids compared to their parental cells. The initial in vitro study showed that EVs and their parental cells can be correctly classified (100% accuracy) between cancerous and non‐cancerous, as well as into their respective breast cancer subtypes, based on their lipid composition. Subsequently, an untargeted lipidomic analysis was carried out for blood plasma EVs from women diagnosed with breast cancer (primary or progressive metastatic breast cancer) as well as healthy women. Correspondingly, when blood plasma EVs were analysed, breast cancer patients and healthy women were correctly classified with an overall accuracy of 93.1%, based on the EVs’ lipid composition. Similarly, the analysis of patients with primary breast cancer and healthy women showed an overall accuracy of 95% for their correct classification. Furthermore, primary and metastatic breast cancers were correctly classified with an overall accuracy of 89.5%. This reveals that the blood plasma EVs’ lipids may be a promising source of biomarkers for detection of breast cancer. Additionally, this study demonstrates the usefulness of untargeted lipidomics in the study of EV lipid composition and EV‐associated biomarker discovery studies. This is a proof‐of‐concept study and a starting point for further analysis on the identification of EV‐based biomarkers for breast cancer.

## 1 INTRODUCTION

Breast cancer continues to be the most commonly diagnosed cancer worldwide, and its future burden is predicted to continue to increase in the decades to come (Arnold et al., 2022). Over 2.3 million new breast cancer cases occurred in 2020, with an incidence rate over 80 per 100,000 females in parts of Europe, Northern America and Australia/New Zealand (Arnold et al., 2022). By 2040, the number of new cases is predicted to increase by over 40% (Arnold et al., 2022). Breast cancer can be classified into different molecular subtypes based on the expression of the estrogen receptor (ER), progesterone receptor (PR) and the human epidermal growth factor receptor 2 (HER2, also known as HER2/neu or ErbB2) or the lack of the expression of these receptors (triple‐negative breast cancer, TNBC) (Langlands et al., 2013). Self‐examination, clinical breast exam and imaging are the first steps for a breast cancer diagnosis, but a tissue biopsy using invasive methods is required if malignancy is suspected (Newell & Mahoney, 2014). A liquid biopsy could bring significant benefits in the screening, early diagnosis, prognosis and monitoring of the progression of breast cancer, which subsequently contribute to the patients’ early access to treatment (Alimirzaie et al., 2019; Tay & Tan, 2021; Venetis et al., 2023).

Extracellular vesicles (EVs), including exosomes and microvesicles, are found in body fluids and can be a source of biomarkers for cancer diagnosis (Logozzi et al., 2009, Skotland et al., 2017). Cancer cells as well as immune cells produce EVs, which play an important role in cancer development, progression and metastasis (Bell and Taylor, 2016; Boelens et al., 2014; Tickner et al., 2014). Proteins and microRNA found in EVs have been widely analysed (Kalra et al., 2012, Keerthikumar et al., 2016). In contrast, their lipid content remains underexplored. The lipid composition of cancer‐derived EVs has mainly been studied by analysing melanoma, colorectal, breast and prostate cancer cell lines (Brzozowski et al., 2018; Hosseini‐Beheshti et al., 2012; Llorente et al., 2013; Lobasso et al., 2021; Lydic et al., 2015; Nishida‐Aoki et al., 2020; Skotland et al., 2017). In breast cancer, a lipidomic study of two metastatic TNBC cell lines found enrichment of unsaturated diacylglycerols in EVs produced by high‐metastatic cells compared to EVs produced by low‐metastatic cells (Nishida‐Aoki et al., 2020). Lipidomic analysis of urinary EVs from prostate cancer patients has suggested that a combination of two phosphatidylserines (PS) and one lactosylceramide (LacCer) can distinguish between healthy individuals and prostate cancer patients; PS(18:1/18:1), PS(18:0_18:2) and LacCer(d18:1/16:0) (Skotland et al., 2017). This demonstrates the importance of lipidomic analysis of EVs found in cancer patients’ body fluids and its potential for the identification of lipid biomarkers for cancer diagnosis.

Lipidomic analysis using mass spectrometry (MS)‐based approaches such as liquid chromatography‐MS (LC‐MS) and imaging MS allow a comprehensive study of lipid alterations in cancer, including breast cancer. High levels of sphingolipids have been detected in breast cancer tissue when compared to normal breast tissue, using LC‐MS‐based approaches (Nagahashi et al., 2016). Similarly, differences in the lipid profile between breast cancer subtypes have been found by analysing breast cancer cell lines (Eiriksson et al., 2020). In addition, MS‐based technologies using matrix‐assisted laser desorption/ionization (MALDI) (Kang et al., 2011), desorption electrospray ionisation (DESI) (Guenther et al., 2015) and rapid evaporative ionization mass spectrometry (REIMS) (St John et al., 2017) have demonstrated to be robust technologies for breast cancer detection, thanks to the high sensitivity and relative straightforwardness of studying the lipid content of cancerous tissues by MS.

EV‐based liquid biopsy could overcome the limitations associated with tissue biopsy; this includes access to tumour tissue and invasiveness, as well as tumour heterogeneity (Armakolas et al., 2023; Liang et al., 2021; Martins et al., 2021). Lipidomic analysis of EVs circulating in cancer patients’ body fluids combines the robustness and sensitivity of MS‐based technologies for cancer detection with the clinical advantage of using non‐ or minimally invasive methods for sample collection. This approach has great potential not only for cancer diagnosis, but also for cancer prognosis, monitoring and stratified medicine. In this proof‐of‐concept study, we studied the lipid composition of EVs produced by breast cancer cell lines from a range of molecular subtypes and the potential of EVs found in breast cancer patients’ blood plasma for the identification of lipid biomarkers for breast cancer detection. We carried out an untargeted lipidomic analysis of a comprehensive panel of cancerous and non‐cancerous mammary epithelial cells and their secreted EVs, as well as EVs found in blood plasma from breast cancer patients and healthy women. To date, this approach has not been explored.

## 2 MATERIALS AND METHODS

### 2.1 Mammary epithelial cells cultures

Nine breast cancer cell lines were studied: two ER+/PR+ (MCF‐7 and T47D), two HER2+ (HCC1954 and JIMT‐1) and five TNBC cell lines (MDA‐MB‐436, MDA‐MB‐231, MDA‐MB‐468, HCC1937 and Hs578T). In addition, non‐cancerous mammary epithelial cells (MCF‐10A and HuMEC) were also analysed. The cells were authenticated using Single Tandem Repeat analysis (STR), except HuMEC which was obtained from Thermo Fisher Scientific (MA, USA). All cells except MCF‐10A and HuMEC were cultured in high glucose Dulbecco's Modified Eagle's Medium (DMEM) supplemented with 10% fetal bovine serum, 1x penicillin/streptomycin, 20 mM HEPES and 1x non‐essential amino acids (all from Thermo Fisher Scientific). DMEM/F12 medium supplemented with 5% (v/v) horse serum, 1x penicillin/streptomycin (all from Thermo Fisher Scientific), 20 ng/mL epidermal growth factor (PeproTech, NJ, USA), 10 µg/mL insulin, 100 ng/mL cholera toxin and 0.5 µg/mL hydrocortisone (all from Sigma–Aldrich, MO, USA) was used to culture MCF‐10A cells. HuMEC cells were maintained in HuMEC Ready Medium (Thermo Fisher Scientific). Cells were maintained at 37°C in 5% CO_2_. Experiments for each cell line were performed in triplicate.

### 2.2 Isolation of EVs from conditioned medium

EV isolation was performed as described previously (Horgan et al., 2020; Penders et al., 2021). Cells were expanded in 10x T225 cell culture flasks and once cells reached ∼80%–90% confluency, they were maintained in their respective supplemented medium in the absence of serum for 72 h. For HuMEC Ready Medium (serum‐free), the bovine pituitary extract was omitted due to presence of abundant particulates. Conditioned media was harvested, filtered with a 0.45 µm membrane bottle‐top filter, and stored at −80°C until further processing. The conditioned media were concentrated using 100 kDa MWCO Amicon Ultra‐15 centrifugal filter devices (Sigma–Aldrich) and centrifugation at 5000 x g at 4°C to a final volume of 500 µL. Subsequently, EVs were isolated by size exclusion chromatography using a Sepharose CL‐2B (Sigma–Aldrich) column (1 cm × 30 cm) packed to approximately 28 cm and equilibrated using particle‐free Dulbecco's Phosphate‐Buffered Saline (DPBS, Thermo Fisher Scientific). Concentrated conditioned media was loaded onto the column and DPBS was used as the mobile phase. Fractions of 1 mL were eluted by gravity and the peak fractions containing the EVs (fractions 8–12) were pooled and stored at −80°C until further analyses.

### 2.3 Whole blood collection

Before surgery was undertaken, blood samples were collected from ten women with primary breast cancer (age range 36–68 years and median age 52 years). All primary breast cancer samples, except one, were obtained from treatment‐naive patients. Blood samples were obtained from an additional nine women with progressive metastatic breast cancer (age range 36–72 years and median age 53 years), who were under cancer treatment but not responding to the treatment. In addition, samples were obtained from ten healthy women who neither had pre‐existing medical conditions and infections at the time the samples were collected or in the previous weeks (age range 36–62 years and median age 50 years). Whole blood samples were collected under fasting conditions by venepuncture using EDTA (K2E) vacutainer tubes. Blood plasma was obtained by 10 min centrifugation at 1000 x g at 4°C, followed by the collection of the supernatant and its centrifugation in a new tube for 10 min at 2000 x g at 4°C. The blood plasma obtained was stored at −80°C until further analyses.

### 2.4 Ethics approval

Human samples used in this research project were obtained from the Imperial College Healthcare Tissue Bank (ICHTB). Tissue Bank projects Ref R17023, R11015 and R16056. ICHTB is supported by the National Institute for Health Research (NIHR) Biomedical Research Centre based at Imperial College Healthcare NHS Trust and Imperial College London. ICHTB is approved by Wales REC3 to release human material for research (22/WA/0214). All women participated voluntarily, and their personal data was kept anonymous.

### 2.5 Isolation of EVs from human blood plasma

The blood plasma samples were centrifuged at 5000 x g for 15 min at 4°C in order to remove cell debris, if still present. The supernatant was transferred into a new tube and centrifuged at 16,000 x g for 30 min at 4°C to remove larger microvesicles. The supernatant was then transferred into a new tube, and it was kept at 4°C to be used the same day. Iodixanol density gradients were prepared using OptiPrep (60%, w/v, Sigma–Aldrich) according to the manufacture's recommendations. Initially, a working solution of 50% OptiPrep was obtained by mixing five volumes of 60% OptiPrep with one volume of a homogenisation solution, consisting of 0.25 M sucrose, 6 mM EDTA and 6 mM Tris‐HCl at pH 7.4. The OptiPrep working solution was diluted to 30% and 6%, in a buffer solution consisting of 0.25 M sucrose, 1 mM EDTA and 1 mM Tris‐HCl at pH 7.4. One mL of 30% and 1 mL of 6% OptiPrep were layered in ultra‐clear centrifuge tubes (Beckman Coulter, CA, USA) from bottom to top. This was followed by 3 mL of the blood plasma which was clear of debris and larger microvesicles. The samples were centrifuged in a Beckman Coulter's SW 55 Ti swinging‐bucket rotor for 2 h at 120,000 x g_avg_ at 4°C, setting up the acceleration to maximum and deceleration to zero. The centrifugation tubes were carefully removed from the rotor and ten 500 µL fractions were collected from top to bottom. The fractions nine and ten, containing the EVs (but also lipoproteins with the same density, mainly High‐Density Lipoproteins (HDL)) were added on top of a chromatography column (Bio‐Rad, CA, USA) containing 2 mL of Capto Core 700 (Sigma–Aldrich), which combines size separation and binding chromatography to deplete co‐isolation of non‐EV components. Once the mix of the two fractions containing EVs entered the stationary phase completely, DPBS was used as the mobile phase and three 1 mL fractions were collected by gravity for EVs’ analysis, pooled and stored at −80°C until further analyses.

### 2.6 Density gradient measurement

The absorbance of 6%, 10%, 20% and 30% iodixanol solutions (OptiPrep) were measured at 340 nm by spectrophotometry (as suggested by manufacturer) and a standard curve was created. The absorbance of each fraction collected was also measured and their density calculated based on the iodixanol standard curve.

### 2.7 Nanoparticle tracking analysis

EVs’ concentration was measured using a NanoSight NS300 (Malvern Panalytical Ltd, England, United Kingdom) equipped with a 532 nm laser and a sCMOS camera. EVs were diluted in particle‐free DPBS to a concentration of 1–10 × 10^8^ particles/mL. Five 60 s videos were analysed of the diluted EVs using NTA V3.0 software.

### 2.8 Protein quantification

Protein concentration analysis was carried out using the Pierce BCA protein assay kit (Thermo Fisher Scientific) according to manufacturers’ instructions. Samples were incubated at 37°C for 30 min and absorbance was measured at 562 nm.

### 2.9 Immunoblotting analyses

A pooled sample of EVs isolated from blood plasma and a pooled sample of blood plasma were analysed using western blotting. Proteins were extracted from the pooled samples by adding RIPA lysis buffer containing protease and phosphatase inhibitors and sonication (mode pulse) for 20 s in ice‐water. Samples were mixed for 1 h at 4°C and then centrifuged at 20,000 x g for 10 min at 4°C. Supernatants (10 µL) were used for protein concentration analysis using the BCA protein assay kit (Thermo Fisher Scientific). Protein extracts (15 µg) were mixed with Laemmli sample buffer (Bio‐Rad) without reducing agent, loaded and separated on 4%–20% Mini‐PROTEAN TGX precast protein gels (Bio‐Rad). Proteins were transferred to polyvinylidene fluoride (PVDF) membranes (Bio‐Rad) and blocked in 5% (w/v) non‐fat dry milk (Bio‐Rad) in TBS‐T (Tris‐Buffered Saline with 0.1% (v/v) Tween‐20 (Sigma–Aldrich)) for 1 h at room temperature, followed by three 10 min washes in TBS‐T. Three primary antibodies were evaluated: mouse anti‐CD9 (Cat#10626D, RRID:AB_2532982), anti‐CD81 (Cat#10630D, RRID:AB_2532984) and anti‐apopoliprotein A1 (ApoA1, Cat#MIA1405, RRID:AB_11152905), all from Thermo Fisher Scientific. The membranes were incubated overnight at 4°C in one of the primary antibodies diluted 1:1000 in 5% (w/v) bovine serum albumin (Sigma–Aldrich). Subsequently, membranes were washed three times in TBS‐T and incubated for 1 h at room temperature with secondary HRP‐linked antibody against mouse IgG (1:2000 in blocking buffer, Cell Signaling Technology (MA, USA) Cat#7076, RRID:AB_330924). After additional washing in TBS‐T three times, the membranes were scanned using the LAS‐3000 Imaging system (Fujifilm, Japan).

### 2.10 Transmission electron microscopy (TEM)

EVs isolated from blood plasma were analysed using TEM by simple drop casting. In one typical preparation, 1 µL of sample was drop casted directly onto a copper grid (Carbon support film square grid, 400 mesh, 5–6 nm, copper, Electron Microscopy Sciences) and allowed to dry at room temperature overnight. Dried samples were then imaged with a JEOL 2100Plus (LEOL, Japan) transmission electron microscope at 200 kV.

### 2.11 Lipid extraction

Lipids were extracted from EVs and cells based on the Bligh and Dyer method (Bligh & Dyer, 1959). To summarise, 3.75 mL of chloroform:methanol (1:2, v/v) were added to 1 mL of the sample homogenized in LC‐MS grade water and vortexed. Samples were processed on ice and incubated for 30 min for the extraction of the lipids into the organic phase. After the incubation time, 1.25 mL of chloroform was added, and the samples were vortexed. Next, 1.25 mL of LC‐MS grade water was added and the mixture vortexed again. The samples were centrifuged at 1000 x g for 10 min at 4°C to separate the chloroform layer (bottom layer) containing the lipids from the aqueous or methanolic layer (top layer) containing non‐lipids. In parallel, ‘blank samples’ were prepared by replacing the sample by 1 mL of LC‐MS grade water to allow a washing step to be included and obtain a much cleaner sample of lipids. The washing step consisted of interchanging the organic and aqueous layers between the ‘blank samples’ and the samples, and then centrifuging at 1000 x g for 10 min at 4°C. After the final centrifugation, the organic layer was collected and dried using nitrogen. The lipids extracted from mammary epithelial cells and their EVs were reconstituted in acetonitrile/isopropanol (1:1, v/v), the samples were normalised based on the protein concentration estimated from the protein disc obtained during the lipid extraction. Lipids extracted from blood plasma EVs were reconstituted in LC‐MS grade water/isopropanol (1:5, v/v) based on the number of EVs per sample.

### 2.12 UPLC‐MS and UPLC‐MS/MS analyses

Lipids extracted from cells and their released EVs, as well as from EVs isolated from blood plasma, were analysed by Ultra Performance Liquid Chromatography (UPLC)‐MS. An ACQUITY UPLC System coupled to a Waters XEVO G2 Q‐TOF mass spectrometer (Waters Corporation, MA, USA) was used. The liquid chromatographic separation of the compounds was carried out using an ACQUITY UPLC BEH C8 Column (1.7 µm, 2.1 × 100 mm, column temperature 55°C). Mobile phase A consisted of LC‐MS grade water:acetonitrile:isopropanol (2:1:1, v/v/v), 5 mM ammonium acetate, 0.05% acetate acid and 20 µM phosphoric acid. Mobile B consisted of acetonitrile:isopropanol (1:1, v/v), 5 mM ammonium acetate and 0.05% acetic acid. The binary gradient profile for the LC‐MS analyses of the mammary cells and their EVs was 99.9% mobile phase A (0.0–2.0 min), 70% A (2.0–11.5 min), 10% A (11.5–12.0 min), 0.1% A (12.0–12.50 min) and 99.9% A (12.50–14.6 min). LC‐MS analyses of lipids extracted from EVs isolated from blood plasma were carried using the facilities and methodologies defined by the National Phenome Centre from Imperial College London for the untargeted lipidomic analysis of human blood plasma. The binary gradient profile was 99% mobile phase A (0.0–2.0 min), 70% A (2.0–11.5 min), 10% A (11.5–12.0 min), 0.1% A (12.0–12.50 min), 35% A (12.50–12.55 min), 70% A (12.55–12.65 min), 99% A (12.65–12.75 min) and 99% A (12.75–13.25 min). For both, the MS and MS/MS data were acquired for 0.1 s in the centroid mode in both positive electrospray ionization mode (ESI+) and negative electrospray ionization mode (ESI‐). Mass spectrometer source parameters were as follow: capillary voltage 2 kV (ESI+) or 1.5 kV (ESI‐), sampling cone voltage 25 V, source temperature 120°C, desolvation temperature 600°C, cone gas flow 150 (L/h) and desolvation flow 1000 (L/h). Quality Control (QC) samples containing a pool of all the samples evaluated and lipid internal standards were included in the set of samples analysed. This was to assess the quality of the data throughout the whole running time. Data dependent acquisition (DDA) was achieved for QC samples to perform MS/MS experiments to identify lipid species. A collision energy ramp of 22–37 V was applied for the fragmentation of the parent ions for both electrospray ionization modes. In addition, individual MS/MS experiments were also carried out for the relevant lipid species identified.

### 2.13 Data analysis

MS data pre‐processing was undertaken using XCMS open‐source software (Smith et al., 2006). The XCMS outputs included *m/z*, retention time and intensity for each peak detected. Features with coefficients of variation (CV%) in QC samples higher than 30% (non‐reproducible measurements) were removed from the outputs. Sample intensities were normalised by probabilistic quotient normalisation for univariate analysis. For principal components analysis (PCA), the intensities were also log_10_ transformed. Univariate and multivariate analyses were carried out using Python programming language (IPython, RRID:SCR_001658) and the package scikit‐learn (Pedregosa et al., 2011). To identify the lipid species significantly enriched in EVs when compared to their parental cells, log_2_ fold changes were calculated for each detected feature based on the mean of the normalised relative abundances for each group studied (i.e., cells and EVs). A threshold of log_2_ fold > 2 or < −2 was defined for the analysis of the lipid enrichment between EVs and cells, respectively. Statistically significant differences between the means of EVs and cells were calculated by one‐way ANOVA. *P*‐values were adjusted by Benjamini–Hochberg correction (*q*‐values) and a threshold of statistical significance was set at lower than 0.05 (*q*‐value < 0.05). Spearman rank‐order correlation coefficient was calculated for paired cells and their secreted EVs to identify lipids correlated between these two groups (i.e., cells and EVs). Logistic regression classification with recursive feature elimination (LR‐RFE) analysis was performed for the selection of a subset of the most relevant features for the analysis between groups (i.e., cancer and no‐cancer, or between the three types of breast cancer subtypes studied). To evaluate the performance of the LR‐RFE model, leave‐one‐group‐out cross‐validated LR classification was performed for the study of EVs and their parental cells (biological replicates were defined as one group) and leave‐one‐individual‐out cross‐validated LR classification for the study of EVs found in blood plasma. The Area Under the Receiver Operating Characteristic (AUROC) curve was determined to measure the accuracy of the LR classification models generated with a reduced number of features. The LC‐MS datasets obtained by ESI+ and ESI− modes were fused by concatenation, with all variables being auto‐scaled (subtraction of mean and division by standard deviation) for LR‐RFE classification; box plots also show auto‐scaled data. Lipid annotations were performed using in‐house databases and matching them with online lipid databases (LIPID MAPS, HMDB and METLIN) and by the manual verification of MS/MS spectrum. Mann–Whitney U tests were performed to evaluate whether significant differences were observed in the number of EV's isolated from the blood plasma samples of the three groups studied: (i) healthy volunteers, (ii) primary breast cancer and (iii) progressive metastatic breast cancer.

## 3 RESULTS

Untargeted lipidomic analysis using reversed‐phase LC‐MS was carried out for cancerous and non‐cancerous human mammary epithelial cells and their secreted EVs, and subsequently for EVs found in blood plasma from women with breast cancer as well as healthy women. The methodology implemented in this study allowed the identification of a wide range of lipids. This included sphingolipids among them sphingomyelins (SM), ceramides (Cer), and hexosylceramides (HexCer); glycerophospholipids included PS, phosphatidylcholines (PC), ether‐PC, phosphatidylinositols (PI), phosphatidylethanolamines (PE), ether‐PE, phosphatidic acids (PA) phosphatidylglycerols (PG), lysophosphatidic acids (LPA), lysophosphatidylethanolamines (LPE), and lysophosphatidylcholines (LPC); glycerolipids included triglycerides (TG); and fatty acids (FA).

### 3.1 Sphingolipids and glycerophospholipids are the most abundant lipids in breast cancer‐derived EVs

**FIGURE 1:**
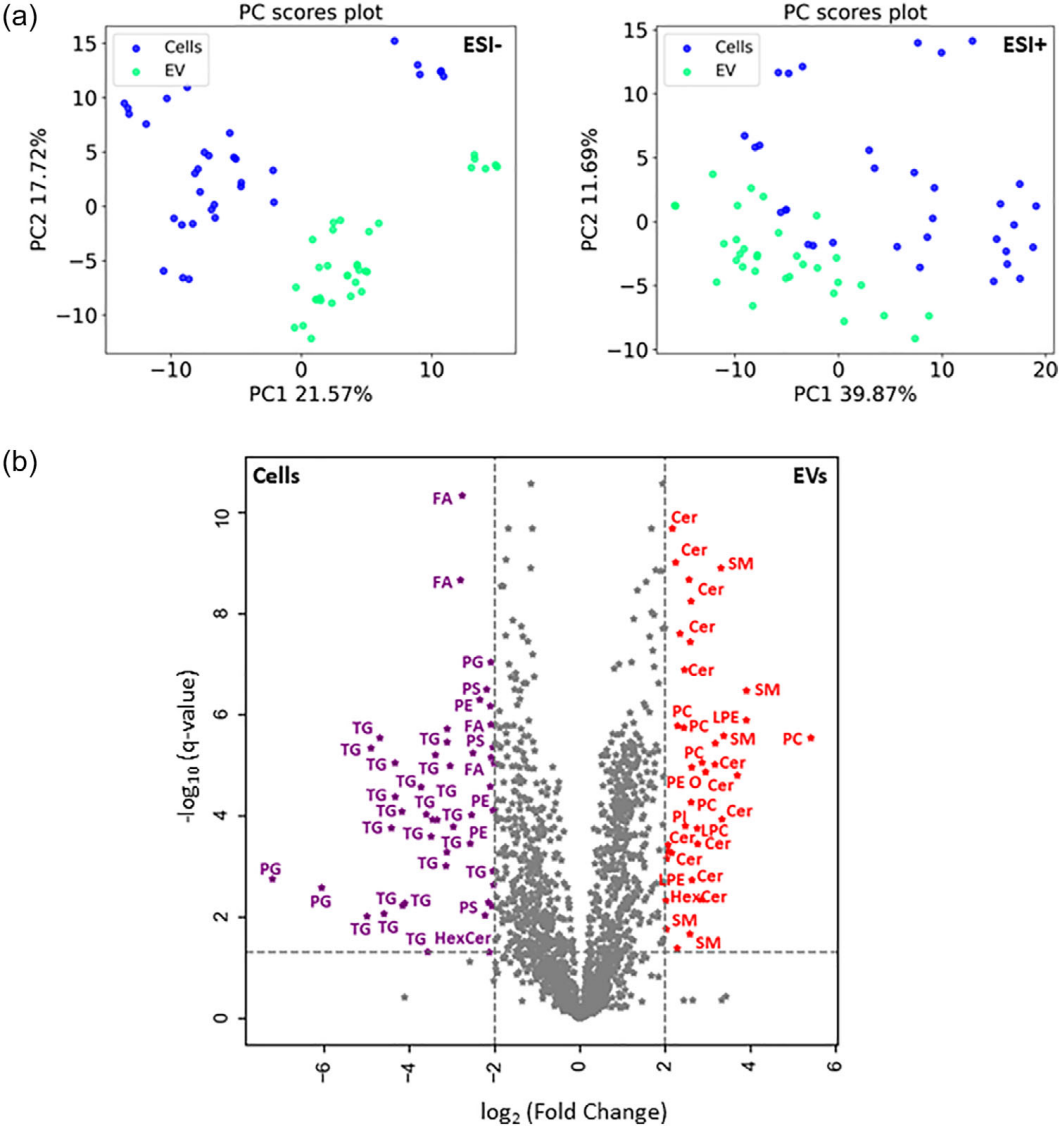
Lipidomic analysis of EVs and their parental cells. (a) PCA showing the LC‐MS data distribution for both cells (*N* = 11 cell lines and 3 biological replicates; *n* = 33) and their secreted EVs (*n* = 33). This includes both cancerous and non‐cancerous cells and their EVs. The LC‐MS data was acquired in both ESI+ and ESI− modes. (b) Volcano plot showing the lipids significantly (*q*‐value < 0.05) enriched in breast cancer‐derived EVs (log_2_ fold change > 2, red stars) when compared to their parental cells (log_2_ fold change < −2, purple stars). *n* = total number of observations.

Initially, an in vitro study was carried out to understand the lipid composition of EVs produced by breast cancer cells. For this, an untargeted lipidomic analysis of a comprehensive panel of cultured cancerous human mammary epithelial cell lines and their secreted EVs was carried out. The cell panel included nine breast cancer cell lines (MCF‐7, T47D, HCC1954, JIMT‐1, MDA‐MB‐436, MDA‐MB‐231, MDA‐MB‐468, HCC1937 and Hs578T) representative of the ER+/PR+, HER2+ and TNBC molecular subtypes. The EVs produced in vitro by the mammary epithelial cells analysed in this study had a vesicular morphology and were positive for the EV marker proteins CD9, CD63 and CD81, as previously reported for this cell panel (Penders et al., 2021). The PCA carried out to understand the distribution of the LC‐MS data in both ESI+ and ESI− modes showed a separation between EVs and cells based on their lipid compositions (Figure 1a). PCA showing the distribution of the LC‐MS for each biological replicate in both ESI+ and ESI− modes is shown in Figure S1. A volcano plot was generated to display the lipids found to be significantly (*q*‐value < 0.05) enriched in breast cancer‐derived EVs (log_2_ fold change > 2), when compared to their parental cells (log_2_ fold change < −2), as depicted in Figure 1b. Sphingolipids (Cer, SM and HexCer) and glycerophospholipids (LPC, LPE, PC, ether‐PE, and PI) were significantly enriched in EVs, when compared to their parental cells (Figure 1b and Table S1). In contrast, cells were mainly enriched in TG, FA and PG when compared to EVs (Figure 1b and Table S2). EVs could be produced by multivesicular bodies, plasma membrane as well as the Golgi apparatus, and consequently do not represent the entirety of cellular components.

To identify lipids which were highly correlated between EVs and their parental cells, the cells and their respective secreted EVs were paired and Spearman rank‐order correlation coefficients calculated (Table S3). Ether‐PC, ether‐PE and HexCer showed a very strong (Spearman r_s _> 0.9) correlation between EVs and their parental cells (Table S3). In addition, PE, PC and PA species were also identified within those lipids that had a very strong (Spearman r_s _> 0.8) correlation between cells and EVs. This suggests that cells and their secreted EVs’ lipid composition are to some extent related and that the lipid arrangement in EVs could be connected to their cellular origin, including their cancerous origin.

### 3.2 EVs and their parental cells can be correctly classified into cancerous and non‐cancerous

**FIGURE 2:**
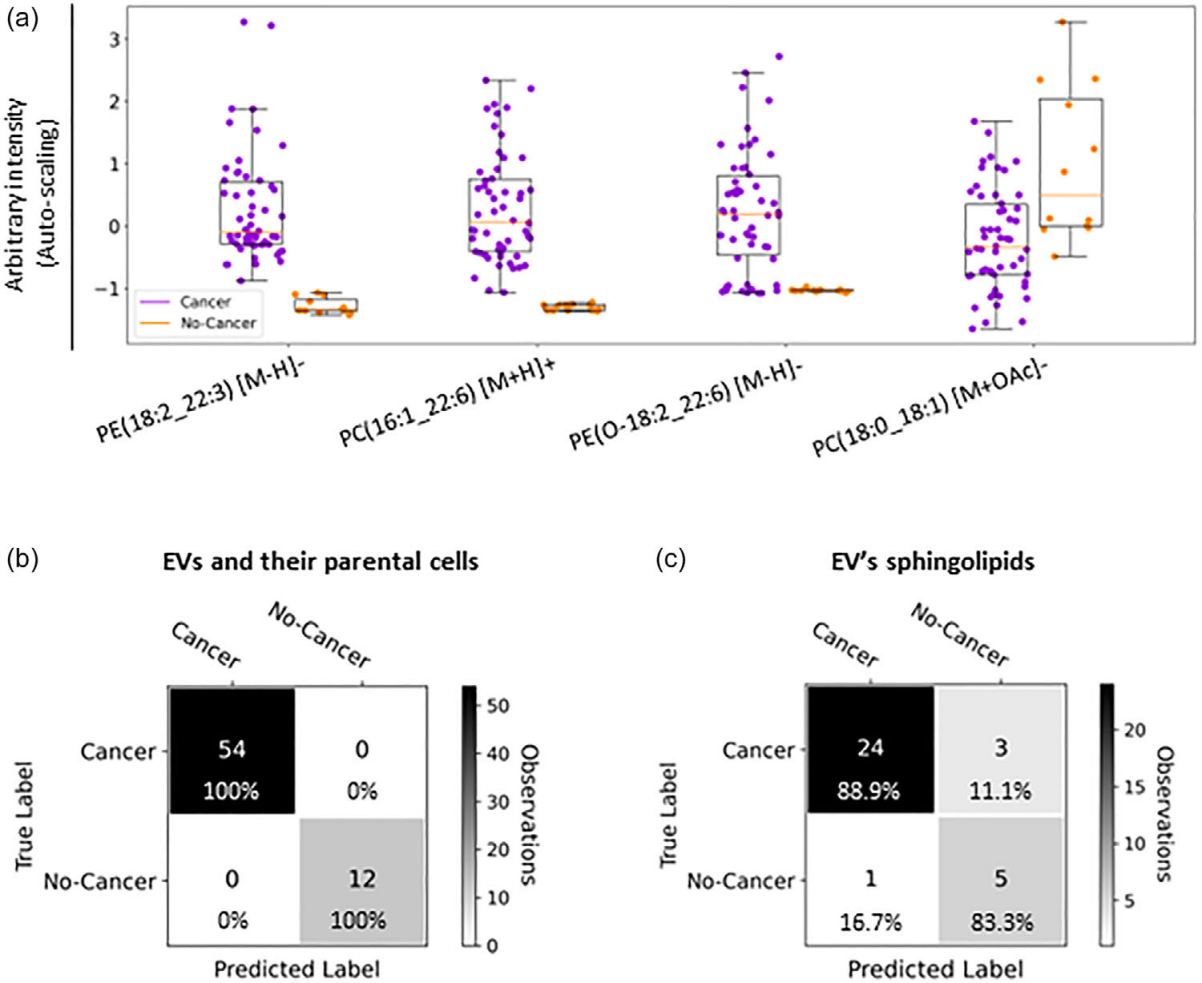
Classification of EVs and cells into cancerous and non‐cancerous. (a) Box plots for the PE and PC species identified by LR‐RFE analysis that distinguish EVs and cells between cancerous (*N* = 9 breast cancer cell lines and their respective EVs, 3 biological replicates; *n* = 54) and non‐cancerous (*N* = 2 non‐cancerous cell lines and their respective EVs, 3 biological replicates; *n* = 12). Box plots are based on the fused and auto‐scaled ESI− and ESI+ datasets. (b) Confusion matrix of the leave‐one‐group‐out cross‐validated LR classification model of EVs and cells into cancerous (*n* = 54) and non‐cancerous (*n* = 12) with an overall accuracy of 100%. (c) Confusion matrix of the leave‐one‐group‐out cross‐validated LR classification model of EVs into cancerous (EVs obtained from *N* = 9 breast cancer cell lines, 3 biological replicates; *n* = 27) and non‐cancerous (EVs obtained from *N* = 2 non‐cancerous cell lines, 3 biological replicates; *n* = 6) based on the sphingolipids found enriched in breast cancer‐derived EVs indicated in Figure 1b and Table S1. The overall accuracy of the model is 87.9%. Biological replicates were defined as one group for leave‐one‐group‐out cross‐validated LR classification analysis. As per the scale on the right‐hand side of the confusion matrix the colour is driven by the number of observations (*n*) rather than the percentages.

To understand whether mammary epithelial cells and their secreted EVs could be classified into cancerous and non‐cancerous, we analysed the lipid composition of cancerous (MCF‐7, T47D, HCC1954, JIMT‐1, MDA‐MB‐436, MDA‐MB‐231, MDA‐MB‐468, HCC1937 and Hs578T) and non‐cancerous human mammary epithelial cells (MCF‐10A and HuMEC) as well as their secreted EVs. The LC‐MS data from the ESI+ and ESI− modes were fused and auto‐scaled to ensure equal contribution of features in both datasets. An LR‐RFE analysis for the fused and auto‐scaled LC‐MS datasets allowed us to identify PE and PC species which allowed EVs and cells to be correctly classified into cancerous or non‐cancerous (Figure 2a,b). The relevant lipid species included PE(18:2_22:3) [M‐H]−, PC(16:1_22:6) [M+H]+, PE(O‐18:2_22:6) [M‐H]− and PC(18:0_18:1) [M+OAc]−. The leave‐one‐group‐out cross‐validated LR classification, carried out to evaluate the performance of the LR‐RFE model, showed that EVs and cells can be classified into cancerous or non‐cancerous with 100% accuracy, based on those lipid species. In addition, ROC analysis based on the combination of these four relevant lipid species showed an outstanding AUROC value equal to 1, indicating the ability of these PE and PC species to distinguish EVs and cells between cancerous and non‐cancerous. Equally, an AUROC equal to 1 can be obtained for PE(18:2_22:3) and PC(16:1_22:6) when analysed separately, indicating these are the main drivers of the classification of EVs and cells into cancerous and non‐cancerous. Additionally, a leave‐one‐group‐out cross‐validated LR classification analysis showed that the EVs secreted by the non‐cancerous and cancerous mammary epithelial cells can be correctly classified with an overall accuracy of 87.9% (Figure 2c), based on the sphingolipids found enriched in breast cancer‐derived EVs when compared to their parental cells (Figure 1b, Table S1 and Figure S2).

### 3.3 EVs and their parental cells can be correctly classified into their respective breast cancer subtype

**FIGURE 3:**
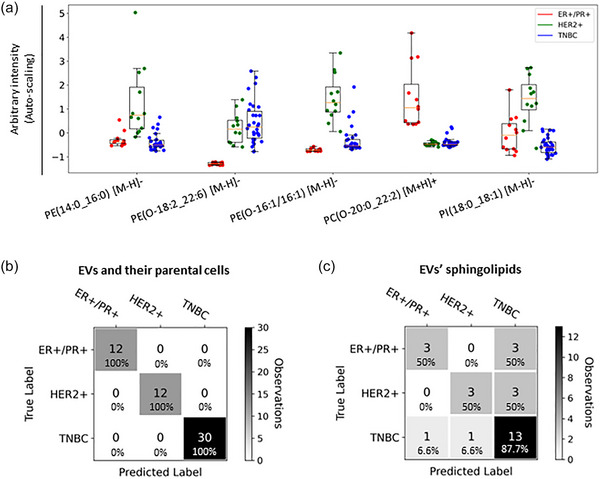
Classification of breast cancer cells and their secreted EVs into their respective breast cancer subtypes. (a) Box plots for the five lipid species identified by the LR‐RFE analysis that classifies breast cancer cells and their EVs into their respective breast cancer subtypes. Nine breast cancer cell lines were studied from different molecular subtypes: ER+/PR+ (*N* = 2 cell lines and their respective EVs, 3 biological replicates; *n* = 12), HER2+ (*N* = 2 cell lines and their respective EVs, 3 biological replicates; *n* = 12), and TNBC (*N* = 5 cell lines and their respective EVs, 3 biological replicates; *n* = 30). Box plots are based on the fused and auto‐scaled ESI− and ESI+ datasets. (b) Confusion matrix of the leave‐one‐group‐out cross‐validated LR classification model of EVs and cells into breast cancer subtypes (100% accuracy). (c) Confusion matrix of the leave‐one‐group‐out cross‐validated LR classification of EVs into their respective breast cancer subtype (overall accuracy of 70.4%) based on the sphingolipids enriched in breast cancer‐derived EVs indicated in Figure 1b and Table S1. Biological replicates were defined as one group for cross‐validation analysis. As per the scale on the right‐hand side of the confusion matrix the colour is driven by the number of observations (*n*) rather than the percentages.

For the fused LC‐MS datasets, a LR‐RFE analysis was performed to study whether EVs and their parental cancerous cells could be correctly classified into the three breast cancer subtypes studied; ER+/PR+, HER2+ and TNBC, based on their lipid composition. The LR‐RFE model allowed the identification of phospholipid species contributing to the classification of the cancerous cells and EVs into their respective breast cancer subtypes (Figure 3a). These relevant lipid species included PE(14:0_16:0) [M‐H]−, PE(O‐18:2_22:6) [M‐H]−, PE(O‐16:1/16:1) [M‐H]−, PC(O‐20:0_22:2) [M+H]+ and PI(18:0_18:1) [M‐H]−. The leave‐one‐group‐out cross‐validated LR classification, showed a perfect classification (100% accuracy) of the EVs and cells according to their respective breast cancer subtype (Figure 3b). In addition, a leave‐one‐group‐out cross‐validated LR classification analysis showed that breast cancer‐derived EVs can be classified into their respective breast cancer subtype with an overall accuracy of 70.4% (Figure 3c), based on the sphingolipids found enriched in breast cancer‐derived EVs (Figure 1b, Table S1 and Figure S3).

### 3.4 There is potential in the lipid composition of blood plasma EVs for breast cancer detection

**FIGURE 4:**
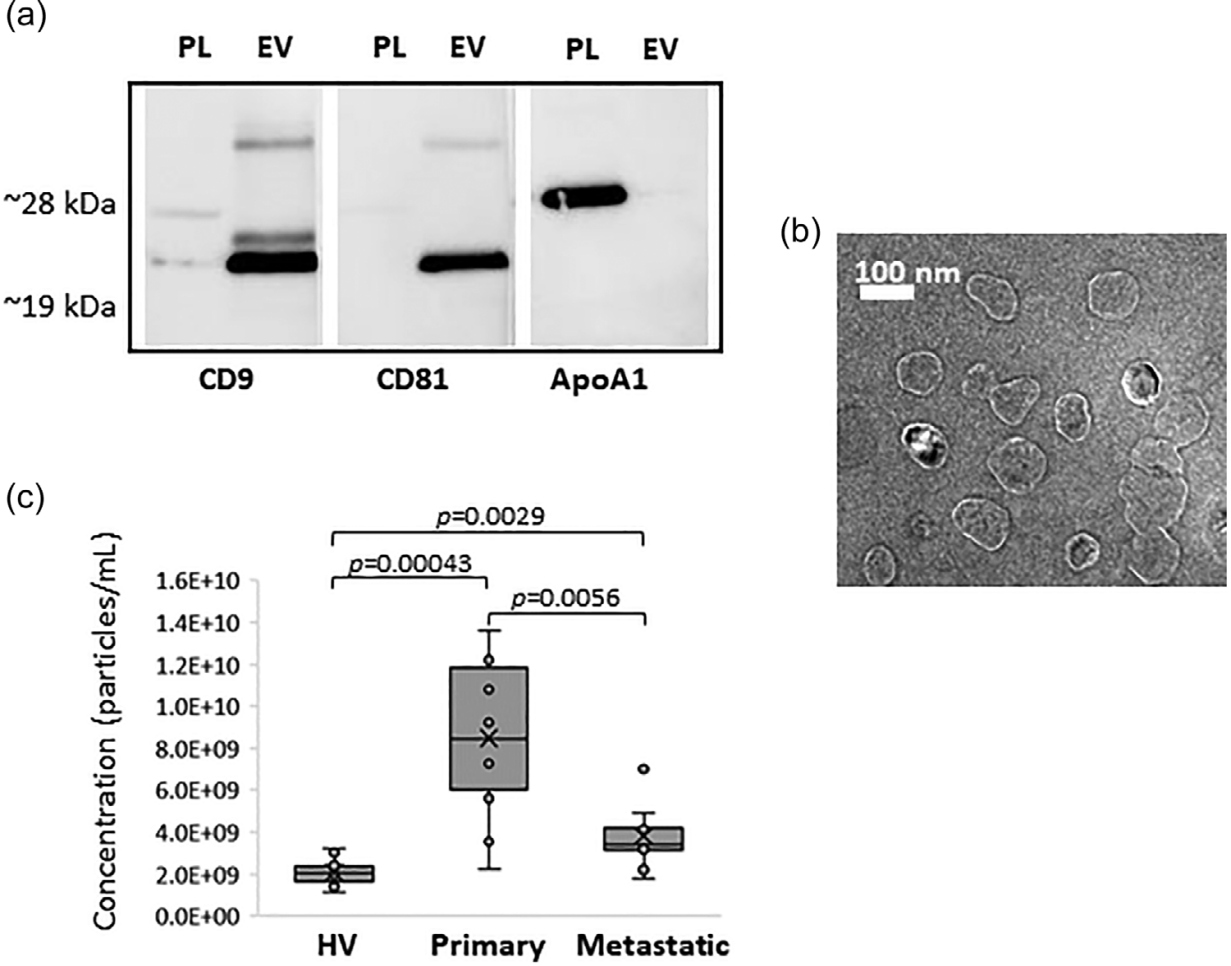
Characterisation of EVs isolated from blood plasma by combination of density gradient ultracentrifugation and size exclusion/bind‐elute chromatography. (a) Western blot analysis of EV protein markers (CD9 and CD81) and ApoA1 in a pooled sample of all blood plasma (PL) samples, and a pooled sample of EVs (EV) isolated from the blood plasma samples studied. (b) EVs morphology analysed by TEM. (c) Box plots for the distribution of the EV concentration per mL obtained from the blood plasma samples from healthy volunteers (HV, *N* = 10), patients with primary breast cancer (Primary, *N* = 10), and patients with progressive metastatic breast cancer (Metastatic, *N* = 9). *P*‐values were obtained by Mann–Whitney U tests for comparisons between the groups studied (HV vs. metastatic, HV vs. primary, and primary vs. metastatic).

Subsequently, we studied EVs isolated from blood plasma from women diagnosed with breast cancer (primary and progressive metastatic breast cancer samples), and healthy women. The blood plasma from breast cancer patients was obtained from patients with invasive ductal carcinoma (IDC), which is the most common histological type of breast cancer, as well as invasive lobular carcinoma (ILC), and ductal carcinoma in situ (DCIS). These samples included hormone receptor‐positive breast cancers (ER+/PR+), HER2+ breast cancers as well as TNBC. Table 1 summarises the pathological characteristics. The EVs isolated from blood plasma were found positive for the EV protein markers CD9 and CD81, and depleted in ApoA1 which is a major protein component of circulating HDL (Figure 4a). These EVs had a density of approximately 1.18–1.23 g/mL and showed a vesicular morphology (Figure 4b). They had an average size of 169.4 nm (mode = 134.9 nm) (Table S4). Significant differences were observed in the number of EVs isolated from blood plasma samples from the healthy volunteers (mean = 2.1E+09 particles/mL) when compared to the primary breast cancer patients (mean = 8.5E+9 particles/mL, *p*‐value < 0.0005) and when compared to the patients with progressive metastatic breast cancer (mean = 3.8E+9 particles/mL, *p*‐value < 0.005) (Figure 4c and Table S4). These results could be attributed to the fact that cancer cells as well as other types of cells triggered by the disease produce EVs, which could contribute to a higher number of EVs circulating in blood from cancer patients. Similarly, significant differences (*p*‐value < 0.05) were observed in the number of EVs isolated from blood plasma samples from primary breast cancer patients when compared to the patients with progressive metastatic breast cancer (Figure 4c and Table S4). The metastatic samples are from breast cancer patients who were under treatment but not responding to the cancer treatment and it is not clear the effect of the treatment in the EVs production from cancer cells and other cells triggered by the disease. Conclusions should not be made based on the number of EVs produced but on their biomolecular composition, EV's cellular origin as well as patients’ clinical information.

**TABLE 1:** Pathological characteristics of breast cancer samples evaluated. Ten samples from patients with primary cancer were evaluated, as well as nine samples from patients with progressive metastatic breast cancer. The information in the table for progressive metastatic breast cancer samples corresponds to the information reported for the initial breast cancer diagnosis (primary cancer).

Cancer	Histological grade	Tumour size (mm)	Histopathology	ER/PR/HER2	Lymph node
Primary	2	20.5	IDC	+/+/−	+
Primary	2	33	ILC	+/+/−	+
Primary	2	21	IDC	+/+/−	–
Primary	1	13	IDC	+/+/−	–
Primary^a^	3	20	IDC	+/+/−	–
Primary	2	25	ILC	+/+/−	–
Primary	1 + 2	10 + 35	IDC	+/+/−	–
Primary	2	72	ILC	+/+/−	–
Primary	2	47	ILC	−/−/−	–
Primary	n.a.	n.a.	DCIS	n.a.	–
Metastatic	2	11	IDC	+/+/−	+
Metastatic	3	44	IDC	+/+/−	+
Metastatic	2	30	IDC	+/+/−	+
Metastatic	3	88	IDC	+/+/−	+
Metastatic	2	17	IDC	−/−/−	–
Metastatic	3	20	IDC	+/+/−	+
Metastatic	2	60	ILC	+/+/−	+
Metastatic	3	7	IDC	−/−/+	+
Metastatic	3	50	IDC	+/+/−	+

Abbreviation: n.a., not available.

^a^ Patient received adjuvant treatment before breast surgery.

**FIGURE 5:**
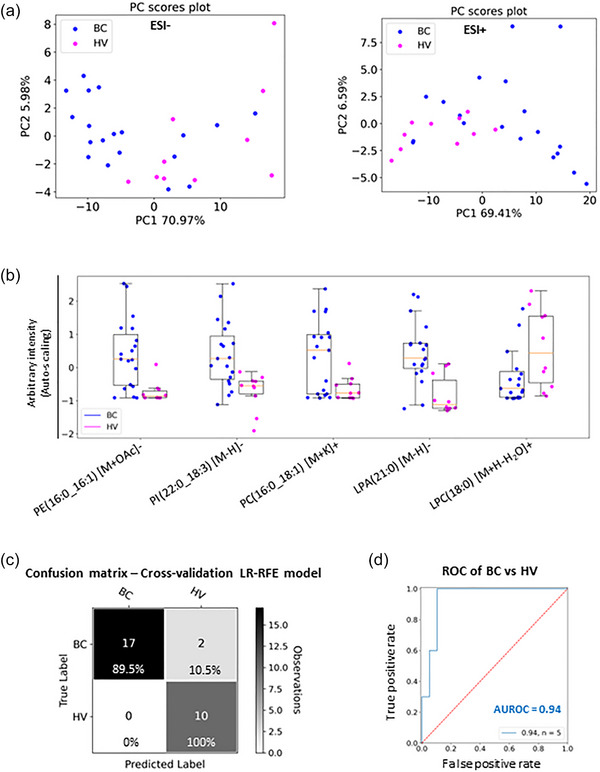
Lipidomic analysis of blood plasma EVs from breast cancer patients and healthy volunteers. (a) PCA showing the LC‐MS data distribution for the breast cancer patients’ samples (BC, *N* = 19), and healthy volunteers (HV, *N* = 10), in both ESI+ and ESI− modes. BC samples include primary (*N* = 10) and progressive metastatic (*N* = 9) breast cancer samples. (b) Box plots for the five lipid species identified by the statistical model obtained by LR‐RFE analysis which distinguish BC samples from samples from HV. Box plots are based on the fused and auto‐scaled ESI− and ESI+ datasets. (c) Confusion matrix of the leave‐one‐individual‐out cross‐validated LR classification of BC and HV samples showing an overall accuracy of 93.1%. The colour scheme of the confusion matrix is driven by the number of observations rather than the percentages. (d) AUROC for the combination of the five relevant lipid species was equal to 0.94.

Untargeted lipidomic analysis was carried out for the EVs isolated from blood plasma from breast cancer patients (*N* = 19) and healthy volunteers (*N* = 10). PCA for the LC‐MS data in both ESI+ and ESI− modes showed a separation between breast cancer samples and samples from healthy women, for most of the samples evaluated (Figure 5a). The LC‐MS data from the ESI+ and ESI− modes were fused and auto‐scaled, and then an LR‐RFE analysis was performed. This analysis allowed the identification of phospholipid species which distinguish breast cancer samples from the samples from healthy volunteers (Figure 5b). Those phospholipids included PE(16:0_16:1) [M+OAc]−, PI(22:0_18:3) [M‐H]−, PC(16:0_18:1) [M+K]+, LPA(21:0) [M‐H]− and LPC(18:0) [M+H‐H_2_O]+. Leave‐one‐individual‐out cross‐validated LR classification showed that EVs from breast cancer patients and healthy volunteers can be correctly classified with an overall accuracy of 93.1% based on those lipid species (Figure 5c). In addition, the combination of those phospholipids showed an AUROC equal to 0.94 (Figure 5d).

**FIGURE 6:**
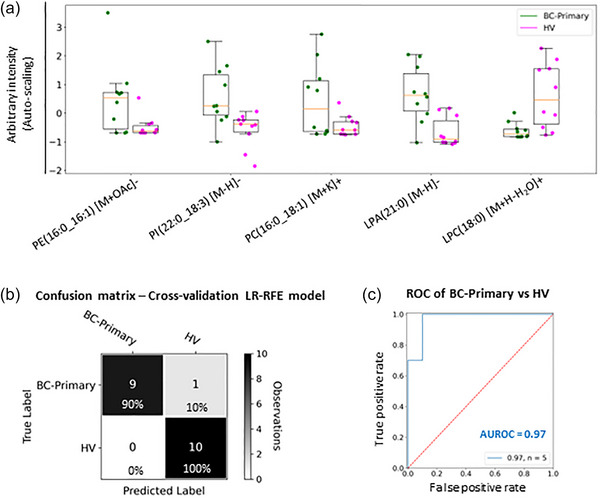
Lipidomic analysis of blood plasma EVs from primary breast cancer patients and healthy volunteers. (a) Box plots for the five lipid species identified by the statistical model obtained by LR‐RFE analysis that distinguish breast cancer patients from healthy volunteers (HV, *N* = 10), but also primary breast cancer patients (BC‐primary, *N* = 10) from HV. Box plots are based on the fused and auto‐scaled ESI− and ESI+ datasets. (b) Confusion matrix of the leave‐one‐individual‐out cross‐validated LR classification of primary breast cancer and HV samples showing an overall accuracy of 95%. The colour scheme of the confusion matrix is driven by the number of observations rather than the percentages. (c) AUROC curve for the combination of the five relevant lipid species was equal to 0.97.

Importantly, the same phospholipids can also distinguish primary breast cancer patients from healthy volunteers (Figure 6a). Leave‐one‐individual‐out cross‐validation analysis showed that samples from primary breast cancer patients (*N* = 10) and healthy volunteers (*N* = 10) can be correctly classified with an overall accuracy of 95% (Figure 6b). Similarly, a ROC analysis showed an AUROC equal to 0.97 for the combination of the five phospholipids (Figure 6c). The primary breast cancer samples included samples from early‐stage breast cancer patients; negative lymph nodes and DCIS (Table 1).

### 3.5 Classification of primary and metastatic breast cancers based on blood plasma EVs’ lipid composition

**FIGURE 7:**
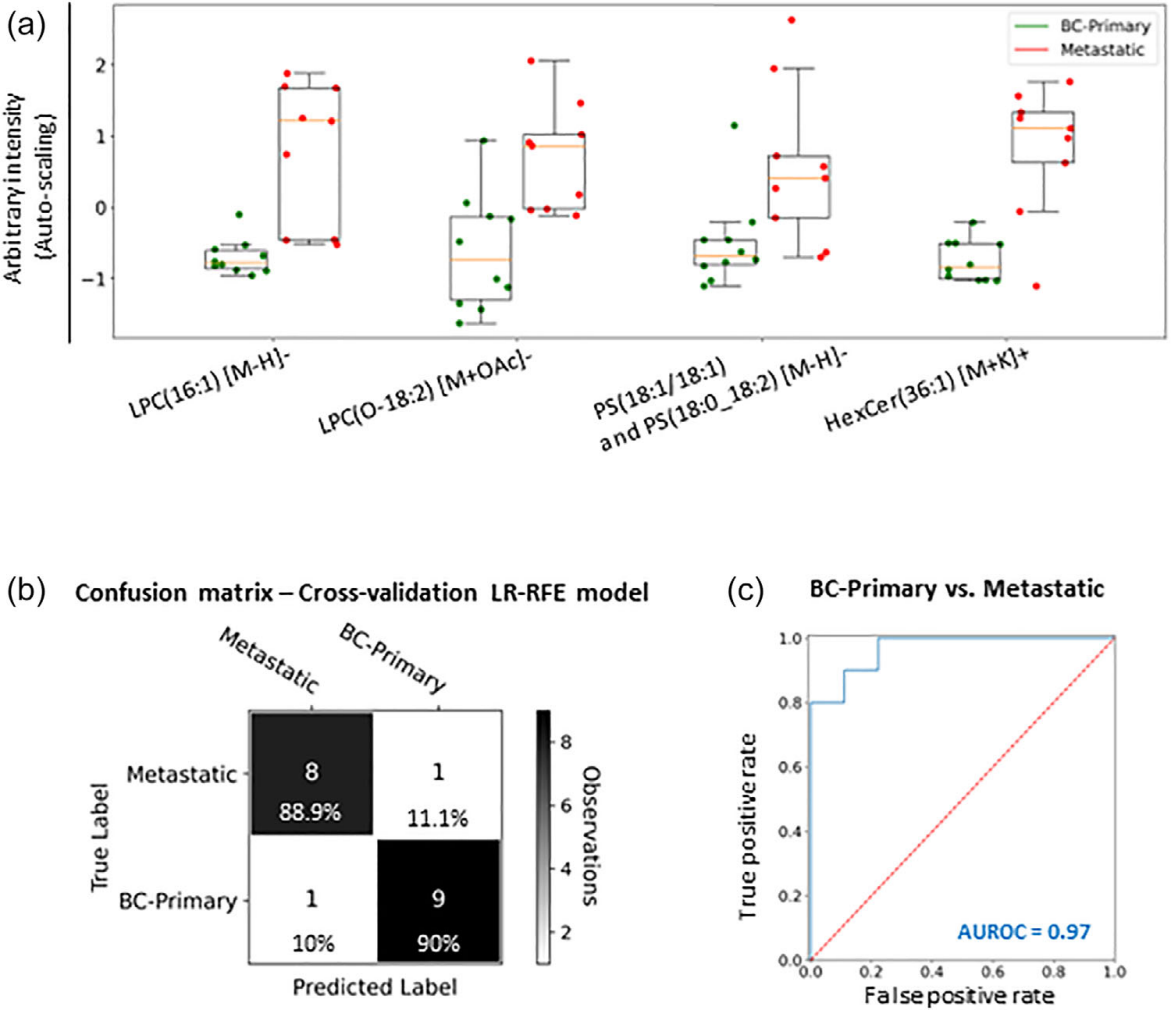
Lipidomic analysis of blood plasma EVs from primary and metastatic breast cancer patients. (a) Box plots for the five lipid species identified by the statistical model obtained by LR‐RFE analysis that distinguishes primary cancers (*N* = 10) from metastatic cancers (*N* = 9). Box plots are based on the fused and auto‐scaled ESI− and ESI+ datasets. (b) Confusion matrix of the leave‐one‐individual‐out cross‐validated LR classification of primary breast cancers and metastatic cancers showing an overall accuracy of 89.5%. The colour scheme of the confusion matrix is driven by the number of observations rather than the percentages. (c) AUROC curve for the combination of the five relevant lipid species was equal to 0.97.

A LR‐RFE analysis showed that blood plasma EV's phospholipid and ceramide species (LPC(16:1) [M‐H]−, LPC(O‐18:2) [M+OAc]−, PS(18:1/18:1) [M‐H]−, PS(18:0_18:2) [M‐H]− and HexCer(36:1) [M+K]+) allow us to distinguish between primary and metastatic breast cancers (Figure 7a). The leave‐one‐individual‐out cross‐validation analysis for the LR‐RFE model showed that primary and metastatic cancers can be classified with an overall accuracy of 89.5% (Figure 7b). In addition, a ROC analysis showed an AUROC equal to 0.97 for the combination of those phospholipid and ceramide species (Figure 7c).

## 4 DISCUSSION

EV's lipid composition is an underexplored field, including the potential in the lipid composition of EVs circulating in blood for breast cancer diagnosis. We demonstrate that untargeted lipidomics is a useful approach for the study of EV's lipid composition and reveal that there is potential in the lipid composition of EVs found in blood plasma for the identification of lipid biomarkers for breast cancer detection.

Initially, we studied the lipid composition of EVs, and their cancerous parental cell lines and we found that sphingolipids (Cer, SM and HexCer) and glycerophospholipids (LPC, LPE, PC, ether‐PE, and PI) are enriched in EVs produced by breast cancer cells. Similar results have been evidenced in the study of EVs secreted by prostate cancer cells (Brzozowski et al., 2018). High abundances of sphingolipids have also been detected in other cancer‐derived EVs’ studies (Brzozowski et al., 2018; Hosseini‐Beheshti et al., 2012; Skotland et al., 2017), including EVs produced by TNBC cells (Nishida‐Aoki et al., 2020). Ether phospholipids were also identified in EVs, which together with SM could play an important role in cellular signalling as well as in the stability of the EV's lipid bilayer as their role as endogenous antioxidants has been suggested (Ogretmen, 2018; ean & Lodhi, 2018). Ether phospholipids have also been identified in EVs released by prostate cancer cell lines (Llorente et al., 2013) and urinary EVs from prostate cancer patients (Skotland et al., 2017). Similarly, lysophospholipids were identified in breast cancer‐derived EVs, they can act as signalling molecules, but they could also participate in the EV membrane curvature (Fuller & Rand, 2001). Lysophospholipids have also been identified in prostate cancer‐derived EVs (Brzozowski et al., 2018; Llorente et al., 2013) and EVs found in blood plasma (Jakubec et al., 2020).

In contrast to EVs, breast cancer cells were enriched in TG and FA. A high content of TG (Eiriksson et al., 2020) and FA (Guenther et al., 2015) was previously reported in cancer cells. Active cell proliferation is fundamental in cancer development and progression and requires the continuous supply of FA (Koundouros & Poulogiannis, 2020). Arachidonic acid (AA) was one of the FA found to be enriched in cells, when compared to EVs. AA is important in cancer development and activation of the PI3K/AKT/mTOR signalling pathway, whose over‐activation contributes to cell proliferation, growth, and cell migration in tumour cells, including breast cancer (Fruman et al., 2017; Wymann & Schneiter, 2008). Cells and their secreted EVs can differ in their lipid composition, but there are also lipid species correlated between them. Ether phospholipids species showed a very strong correlation between EVs and their parental cells. A high level of ether lipids has been observed in cancer cells and their participation in cell differentiation and signalling pathways has been suggested (Benjamin et al., 2013; Dean & Lodhi, 2018). In addition, PC and PE species also showed a strong correlation between cells and their EVs. PC and PE are fundamental lipids in biological membranes, which include EVs membranes, but they are also a source of signalling molecules. PE is important in cell curvature and fluidity, facilitating cell budding, fission and fusion (van Meer et al., 2008) and the same function could be played in EVs.

Our in vitro study showed that phospholipid species allow correct classification (100% accuracy) of mammary epithelial cells and their secreted EVs based on their cancerous or non‐cancerous origin. In addition, we also found that the EVs produced by the non‐cancerous and cancerous cells can be correctly classified with an overall accuracy of 87.9%, based on the sphingolipid species found enriched in EVs when compared to their parental cancer cells. This reflects the fact that metabolic changes are often found in breast cancer tissue (Kang et al., 2011; Nagahashi et al., 2016). We also found that cancerous mammary epithelial cells and their secreted EVs can be correctly classified into their respective ER+/PR+, HER2+ or TNBC subtypes based on their lipid composition. This is in agreement with other studies that have suggested a differentiation of breast cancer subtypes based on the lipid profile of breast cancer cells (Eiriksson et al., 2020). Although the number of cell lines studied per breast cancer subtype is low, and the test can be underpowered because of this, the results of this in vitro study suggest that cancer‐derived EVs carry metabolic phenotype information from the cancer subtype from which they were originated.

We translated our in vitro approach and findings into the lipidomic analysis of EVs found in blood plasma from breast cancer patients and healthy women. We found that blood plasma EVs’ lipid species allowed us to correctly classify patients with breast cancer and healthy women with an overall accuracy of 93.1%. This included detection of ER/PR+, HER2+ and TNBC from patients with primary or progressive metastatic cancer. Importantly, we found that when analysing only the samples from the primary breast cancer patients and healthy women, the same combination of lipids allowed us to correctly classify the samples with an overall accuracy of 95%. Only one sample out of the ten primary breast cancer samples analysed was misclassified. The patient's clinical history indicates that adjuvant treatment was given to the patient before the breast surgery, which could suggest that the treatment could have had an effect on the EV composition and/or production. It is important to highlight the fact that our lipidomic analysis of plasma EV‐based liquid biopsies allowed correct classification of samples from patients with primary cancer at an early stage (0–2), this includes pre‐invasive breast cancer (DCIS, stage 0) and cancers that had not spread to the lymph nodes. Additionally, we found that primary and progressive metastatic breast cancers can be correctly classified with an overall accuracy of 89.5% based on blood plasma EVs’ phospholipid and ceramide species. These findings demonstrate that EVs circulating in breast cancer patients’ blood are a promising source of lipid biomarkers for breast cancer detection, including primary and metastatic cancers, with potential application in detection of breast cancer at an early stage as well as monitoring its progression.

Among the lipids identified in blood plasma EVs relevant in breast cancer detection we found PC and PE which have also been detected in breast cancer tissues as highly abundant lipids (Ide et al., 2013; Punnonen et al., 1989). PE, PC and PI could act as precursors of second messengers including PA, DG and AA which participate in the activation of signalling pathways including PI3K/AKT/mTOR (Foster, 2009; Fruman et al., 2017; Wymann & Schneiter, 2008). PS was found in higher levels in metastatic breast cancers compared to primary breast cancers. The importance of PS as a marker of tumour cells and metastases has been suggested (Riedl et al., 2011). They have also been identified in prostate cancer‐derived EVs and urinary EVs from prostate cancer patients (Llorente et al., 2013; Skotland et al., 2017). LPA(21:0) was identified as one of the relevant lipids in breast cancer detection. LPA has also been identified in EVs found in human blood plasma in other studies (Jakubec et al., 2020). Similarly, odd‐carbon lipid species, have also been detected in both human blood plasma (Huynh et al., 2019) and EVs found in blood plasma (Jakubec et al., 2020). Odd‐chain fatty acids could be produced endogenously or could be associated with the patients’ diet and/or microbiota‐derived vesicles. The key role of the human microbiome in carcinogenesis is a growing area of scientific focus (Xavier et al., 2020), and it has been suggested it may play a role in the risk of estrogen‐dependent cancers (Plottel & Microbiome, 2011). Interestingly, LPA has been associated with breast cancer metastasis, breast cancer treatment failure and postmenopausal breast cancers (Moolenaar et al., 2004). LPA receptors are abundant in breast cancer and have been associated with activation and upregulation of PI3K/AKT, p38‐MAPK and ERK/MAPK signalling pathways, leading to cancer development and progression (Panupinthu et al., 2010).

EVs circulating in blood from cancer patients provide a snapshot of the complex cellular mechanisms triggered by the disease, this includes not only the release of EVs from cancer cells but also non‐malignant cells like immune cells. For instance, neutrophil‐derived EVs could be detected in blood plasma. It has been suggested that a high neutrophil to lymphocyte ratio is associated with a poor breast cancer prognosis (Gago‐Dominguez et al., 2020). In addition, tumour‐associated neutrophils can act as tumour promotors depending on the microenvironment and this cellular crosstalk can be facilitated by both cancer and neutrophil‐derived EVs (Rubenich et al., 2021). This reinforces the importance of the analysis of EVs found in breast cancer patients’ body fluids, as they are a representative picture of all the molecular and cellular processes involved in cancer. This explains differences that can be found between in vitro and clinical studies. It also demonstrates the importance of appropriate selection of methods of isolation of EVs from blood plasma that deplete the co‐isolation of blood plasma components non‐associated with the EV protein corona. This also applies for direct analysis of blood plasma, as EV‐associated cancer biomarkers could be masked by other major components of blood plasma.

The identification of biomarker candidates for breast cancer detection was beyond the scope of this proof‐of‐concept study. It serves as a starting point for future clinical studies which will include analysis of larger cohorts of breast cancer patients as well as those with benign breast conditions. Such studies should also include the analysis of tumour tissues as well as blood plasma EVs from patients before and after breast surgery, to establish whether there is a direct relationship between the presence of a tumour in the body and EV‐associated cancer biomarkers circulating in the patients’ blood. This analysis of blood plasma EV populations combined with studies of multiple types of cancers will be required for identification of specific EV‐based biomarkers for breast cancer detection.

In conclusion, this proof‐of‐concept study reveals the potential in the lipid composition of EVs found in blood plasma for breast cancer detection. Furthermore, this study demonstrates that untargeted lipidomic analysis is a useful approach for the study of the lipid composition of EVs and for EV‐based biomarker discovery studies.

## AUTHOR CONTRIBUTIONS

**Erika Dorado**: Conceptualization; data curation; formal analysis; investigation; methodology; software; validation; visualization; writing—original draft; writing—review and editing. **M. Luisa Doria**: Conceptualization; formal analysis; investigation; methodology; writing—review and editing. **Anika Nagelkerke**: Conceptualization; investigation; methodology; writing—review and editing. **James S. McKenzie**: Data curation; formal analysis; methodology; software; validation; visualization; writing—review and editing. **Stefania Maneta‐Stavrakaki**: Data curation; validation; writing—review and editing. **Thomas Whittaker**: Methodology; writing—review and editing. **Jeremy Nicholson**: Conceptualization; funding acquisition; resources; supervision; writing—review and editing. **R. Charles Coombes**: Conceptualization; resources; supervision; writing—review and editing. **Molly M. Stevens**: Conceptualization; funding acquisition; resources; supervision; writing—review editing. **Zoltan Takats**: Conceptualization; funding acquisition; resources; supervision; writing—review and editing.

## CONFLICT OF INTEREST STATEMENT

E.D. acknowledges support from Merck KGaA, Darmstadt, Germany, through the STRATiGRAD PhD program at Imperial College London. A.N. and M.M.S. acknowledge support from the GlaxoSmithKline Engineered Medicines Laboratory. M.M.S. acknowledges support from the Rosetrees Trust. These organisations did not play any role in the study design, data collection/analysis, manuscript preparation or publication. This work is the subject of an Imperial College Invention Disclosure application.

## Supporting information

Supporting Information

## Data Availability

The data that support the findings of this study are available in the article and supplementary material, further inquiries can be directed to the corresponding authors.

## References

[jev212419-bib-0001] Alimirzaie, S. , Bagherzadeh, M. , & Akbari, M. R. (2019). Liquid biopsy in breast cancer: A comprehensive review. Clinical Genetics, 95(6), 643–660. 30671931 10.1111/cge.13514

[jev212419-bib-0002] Armakolas, A. , Kotsari, M. , & Koskinas, J. (2023). Liquid biopsies, novel approaches and future directions. Cancers, 15(5), 1579. 36900369 10.3390/cancers15051579 PMC10000663

[jev212419-bib-0003] Arnold, M. , Morgan, E. , Rumgay, H. , Mafra, A. , Singh, D. , Laversanne, M. , Vignat, J. , Gralow, J. R. , Cardoso, F. , Siesling, S. , & Soerjomataram, I. (2022). Current and future burden of breast cancer: Global statistics for 2020 and 2040. Breast (Edinburgh, Scotland), 66, 15–23. 36084384 10.1016/j.breast.2022.08.010 PMC9465273

[jev212419-bib-0004] Bell, E. , & Taylor, M. A. (2016). Functional Roles for Exosomal MicroRNAs in the Tumour Microenvironment. Computational and Structural Biotechnology Journal, 15, 8–13. 27872688 10.1016/j.csbj.2016.10.005 PMC5109280

[jev212419-bib-0005] Benjamin, D. I. , Cozzo, A. , Ji, X. , Roberts, L. S. , Louie, S. M. , Mulvihill, M. M. , Luo, K. , & Nomura, D. K. (2013). Ether lipid generating enzyme AGPS alters the balance of structural and signaling lipids to fuel cancer pathogenicity. PNAS, 110(37), 14912–14917. 23980144 10.1073/pnas.1310894110 PMC3773741

[jev212419-bib-0006] Bligh, E. G. , & Dyer, W. J. (1959). A rapid method of total lipid extraction and purification. Canadian Journal of Biochemistry and Physiology, 37(8), 911–917. 13671378 10.1139/o59-099

[jev212419-bib-0007] Boelens, M. C. , Wu, T. J. , Nabet, B. Y. , Xu, B. , Qiu, Y. , Yoon, T. , Azzam, D. J. , Twyman‐Saint Victor, C. , Wiemann, B. Z. , Ishwaran, H. , & Ter Brugge, P. J. (2014). Exosome transfer from stromal to breast cancer cells regulates therapy resistance pathways. Cell, 159(3), 499–513. 25417103 10.1016/j.cell.2014.09.051 PMC4283810

[jev212419-bib-0008] Brosche, T. , & Platt, D. (1998). The biological significance of plasmalogens in defense against oxidative damage. Experimental Gerontology, 33(5), 363–369. 9762517 10.1016/s0531-5565(98)00014-x

[jev212419-bib-0009] Brzozowski, J. S. , Jankowski, H. , Bond, D. R. , McCague, S. B. , Munro, B. R. , Predebon, M. J. , Scarlett, C. J. , Skelding, K. A , & Weidenhofer, J. (2018). Lipidomic profiling of extracellular vesicles derived from prostate and prostate cancer cell lines. Lipids Health Dis, 17(1), 1–12. 30193584 10.1186/s12944-018-0854-x PMC6128989

[jev212419-bib-0010] Coliva, G. , Lange, M. , Colombo, S. , Chervet, J. P. , Domingues, M. R. , & Fedorova, M. (2020). Sphingomyelins prevent propagation of lipid peroxidation‐LC‐MS/MS evaluation of inhibition mechanisms. Molecules (Basel, Switzerland), 25(8), 1925. 32326262 10.3390/molecules25081925 PMC7221532

[jev212419-bib-0011] Dean, J. M. , & Lodhi, I. J. (2018). Structural and functional roles of ether lipids. Protein Cell, 9(2), 196–206. 28523433 10.1007/s13238-017-0423-5 PMC5818364

[jev212419-bib-0012] Eiriksson, F. F. , Nøhr, M. K. , Costa, M. , Bödvarsdottir, S. K. , Ögmundsdottir, H. M. , & Thorsteinsdottir, M. (2020). Lipidomic study of cell lines reveals differences between breast cancer subtypes. PLoS ONE, 15(4), e0231289. 32287294 10.1371/journal.pone.0231289 PMC7156077

[jev212419-bib-0013] Foster, D. A. (2009). Phosphatidic acid signaling to mTOR: Signals for the survival of human cancer cells. Biochimica Et Biophysica Acta, 1791(9), 949–955. 19264150 10.1016/j.bbalip.2009.02.009 PMC2759177

[jev212419-bib-0014] Fruman, D. A. , Chiu, H. , Hopkins, B. D. , Bagrodia, S. , Cantley, L. C. , & Abraham, R. T. (2017). The PI3K pathway in human disease. Cell, 170(4), 605–635. 28802037 10.1016/j.cell.2017.07.029 PMC5726441

[jev212419-bib-0015] Fuller, N. , & Rand, R. P. (2001). The influence of lysolipids on the spontaneous curvature and bending elasticity of phospholipid membranes. Biophysical Journal, 81(1), 243–254. 11423410 10.1016/S0006-3495(01)75695-0 PMC1301507

[jev212419-bib-0016] Gago‐Dominguez, M. , Matabuena, M. , Redondo, C. M. , Patel, S. P. , Carracedo, A. , Ponte, S. M. , Martínez, M. E , & Castelao, J. E. (2020). Neutrophil to lymphocyte ratio and breast cancer risk: Analysis by subtype and potential interactions. Scientific Reports, 10(1), 13203. 32764699 10.1038/s41598-020-70077-z PMC7413522

[jev212419-bib-0017] Guenther, S. , Muirhead, L. J. , Speller, A. V. , Golf, O. , Strittmatter, N. , Ramakrishnan, R. , Goldin, R. D. , Jones, E. , Veselkov, K. , Nicholson, J. , & Darzi, A. (2015). Spatially resolved metabolic phenotyping of breast cancer by desorption electrospray ionization mass spectrometry. Cancer Research, 75(9), 1828–1837. 25691458 10.1158/0008-5472.CAN-14-2258

[jev212419-bib-0018] Horgan, C. C. , Nagelkerke, A. , Whittaker, T. E. , Nele, V. , Massi, L. , Kauscher, U. , Penders, J. , Bergholt, M. S. , Hood, S. R , & Stevens, M. M (2020). Molecular imaging of extracellular vesicles in vitro via Raman metabolic labelling. Journal of Materials Chemistry B, 8(20), 4447–4459. 32373878 10.1039/d0tb00620c PMC7610785

[jev212419-bib-0019] Hosseini‐Beheshti, E. , Pham, S. , Adomat, H. , & Li, N. (2012). Tomlinson Guns ES. Exosomes as biomarker enriched microvesicles: Characterization of exosomal proteins derived from a panel of prostate cell lines with distinct AR phenotypes. Mol Cell Proteomic, 11(10), 863–885. 10.1074/mcp.M111.014845 PMC3494141 22723089

[jev212419-bib-0020] Huynh, K. , Barlow, C. K. , Jayawardana, K. S. , Weir, J. M. , Mellett, N. A. , Cinel, M. , Magliano, D. J. , Shaw, J. E. , Drew, B. G , & Meikle, P. J. (2019). High‐throughput plasma lipidomics: Detailed mapping of the associations with cardiometabolic risk factors. Cell Chem Biol, 26(1), 71–84. e4. 30415965 10.1016/j.chembiol.2018.10.008

[jev212419-bib-0021] Ide, Y. , Waki, M. , Hayasaka, T. , Nishio, T. , Morita, Y. , Tanaka, H. , Sasaki, T. , Koizumi, K. , Matsunuma, R. , Hosokawa, Y. , & Ogura, H. (2013). Human breast cancer tissues contain abundant phosphatidylcholine(36∶1) with high stearoyl‐CoA desaturase‐1 expression. PLoS ONE, 8(4), e61204. 23613812 10.1371/journal.pone.0061204 PMC3629004

[jev212419-bib-0022] Jakubec, M. , Maple‐Grødem, J. , Akbari, S. , Nesse, S. , Halskau, Ø. , & Mork‐Jansson, A. E. (2020). Plasma‐derived exosome‐like vesicles are enriched in lyso‐phospholipids and pass the blood‐brain barrier. PLoS ONE, 15(9), e0232442. 32956358 10.1371/journal.pone.0232442 PMC7505448

[jev212419-bib-0023] Kalra, H. , Simpson, R. J. , Ji, H. , Aikawa, E. , Altevogt, P. , Askenase, P. , Bond, V. C. , Borràs, F. E. , Breakefield, X. , Budnik, V. , & Buzas, E. (2012). Vesiclepedia: A compendium for extracellular vesicles with continuous community annotation. PLoS Biology, 10(12), e1001450. 23271954 10.1371/journal.pbio.1001450 PMC3525526

[jev212419-bib-0024] Kang, H. S. , Lee, S. C. , Park, Y. S. , Jeon, Y. E. , Lee, J. H. , SY, J. , Park, I. H. , Jang, S. H. , Park, H. M. , Yoo, C. W , & Park, S. H (2011). Protein and lipid MALDI profiles classify breast cancers according to the intrinsic subtype. BMC cancer, 11, 465. 22029885 10.1186/1471-2407-11-465 PMC3218066

[jev212419-bib-0025] Keerthikumar, S. , Chisanga, D. , Ariyaratne, D. , Al Saffar, H. , Anand, S. , Zhao, K. , Samuel, M. , Pathan, M. , Jois, M. , Chilamkurti, N. , & Gangoda, L. (2016). ExoCarta: A web‐based compendium of exosomal cargo. Journal of Molecular Biology, 428(4), 688–692. 26434508 10.1016/j.jmb.2015.09.019 PMC4783248

[jev212419-bib-0026] Koundouros, N. , & Poulogiannis, G. (2020). Reprogramming of fatty acid metabolism in cancer. British Journal of Cancer, 122(1), 4–22. 31819192 10.1038/s41416-019-0650-z PMC6964678

[jev212419-bib-0027] Langlands, F. E. , Horgan, K. , Dodwell, D. D. , & Smith, L. (2013). Breast cancer subtypes: Response to radiotherapy and potential radiosensitisation. BJR, 86(1023), 20120601. 23392193 10.1259/bjr.20120601 PMC3608055

[jev212419-bib-0028] Liang, Y. , Lehrich, B. M. , Zheng, S. , & Lu, M. (2021). Emerging methods in biomarker identification for extracellular vesicle‐based liquid biopsy. Journal of Extracellular Vesicles, 10(7), e12090. 34012517 10.1002/jev2.12090 PMC8114032

[jev212419-bib-0029] Llorente, A. , Skotland, T. , Sylvänne, T. , Kauhanen, D. , Róg, T. , Orlowski, A. , Vattulainen, I. , Ekroos, K. , & Sandvig, K. (2013). Molecular lipidomics of exosomes released by PC‐3 prostate cancer cells. Biochimica et Biophysica Acta, 1831(7), 1302–1309. 24046871 10.1016/j.bbalip.2013.04.011

[jev212419-bib-0030] Lobasso, S. , Tanzarella, P. , Mannavola, F. , Tucci, M. , Silvestris, F. , Felici, C. , Ingrosso, C. , Corcelli, A. , & Lopalco, P. (2021). A lipidomic approach to identify potential biomarkers in exosomes from melanoma cells with different metastatic potential. Frontiers in Physiology, 12, 748895. 34867454 10.3389/fphys.2021.748895 PMC8637280

[jev212419-bib-0031] Logozzi, M. , De Milito, A. , Lugini, L. , Borghi, M. , Calabrò, L. , Spada, M. , Perdicchio, M. , Marino, M. L. , Federici, C. , Iessi, E. , & Brambilla, D. (2009). High levels of exosomes expressing CD63 and caveolin‐1 in plasma of melanoma patients. PLoS ONE, 4(4), e5219. 19381331 10.1371/journal.pone.0005219 PMC2667632

[jev212419-bib-0032] Lydic, T. A. , Townsend, S. , Adda, C. G. , Collins, C. , Mathivanan, S. , & Reid, G. E. (2015). Rapid and comprehensive ‘shotgun’ lipidome profiling of colorectal cancer cell derived exosomes. Methods (San Diego, Calif.), 87, 83–95. 25907253 10.1016/j.ymeth.2015.04.014 PMC4615275

[jev212419-bib-0033] Martins, I. , Ribeiro, I. P. , Jorge, J. , Gonçalves, A. C. , Sarmento‐Ribeiro, A. B. , Melo, J. B. , & Carreira, I. M. (2021). Liquid biopsies: Applications for cancer diagnosis and monitoring. Genes, 12(3), 349. 33673461 10.3390/genes12030349 PMC7997281

[jev212419-bib-0034] Moolenaar, W. H. , van Meeteren, L. A. , & Giepmans, B. N. (2004). The ins and outs of lysophosphatidic acid signaling. BioEssays, 26(8), 870–881. 15273989 10.1002/bies.20081

[jev212419-bib-0035] Nagahashi, M. , Tsuchida, J. , Moro, K. , Hasegawa, M. , Tatsuda, K. , Woelfel, I. A. , Takabe, K. , & Wakai, T. (2016). High levels of sphingolipids in human breast cancer. Journal of Surgical Research, 204(2), 435–444. 27565080 10.1016/j.jss.2016.05.022 PMC5002890

[jev212419-bib-0036] Nakajima, M. , Nagahashi, M. , Rashid, O. M. , Takabe, K. , & Wakai, T. (2017). The role of sphingosine‐1‐phosphate in the tumor microenvironment and its clinical implications. Tumour Biology: The Journal of the International Society for Oncodevelopmental Biology and Medicine, 39(4), 1010428317699133. 28381169 10.1177/1010428317699133

[jev212419-bib-0037] Newell, M. S. , & Mahoney, M. C. (2014). Ultrasound‐guided percutaneous breast biopsy. Techniques in Vascular and Interventional Radiology, 17(1), 23–31. 24636328 10.1053/j.tvir.2013.12.005

[jev212419-bib-0038] Nishida‐Aoki, N. , Izumi, Y. , Takeda, H. , Takahashi, M. , Ochiya, T. , & Bamba, T. (2020). Lipidomic analysis of cells and extracellular vesicles from high‐ and low‐metastatic triple‐negative breast cancer. Metabolites, 10(2), 67. 32069969 10.3390/metabo10020067 PMC7073695

[jev212419-bib-0039] Ogretmen, B. (2018). Sphingolipid metabolism in cancer signalling and therapy. Nature Reviews Cancer, 18(1), 33–50. 29147025 10.1038/nrc.2017.96 PMC5818153

[jev212419-bib-0040] Panupinthu, N. , Lee, H. Y. , & Mills, G. B. (2010). Lysophosphatidic acid production and action: Critical new players in breast cancer initiation and progression. British Journal of Cancer, 102(6), 941–946. 20234370 10.1038/sj.bjc.6605588 PMC2844037

[jev212419-bib-0041] Pedregosa, F. , Varoquaux, G. , Gramfort, A. , Michel, V. , Thirion, B. , Grisel, O. , Blondel, M. , Prettenhofer, P. , Weiss, R. , Dubourg, V. , & Vanderplas, J. (2011). Scikit‐learn: Machine learning in python. JMLR, 12, 2825–2830.

[jev212419-bib-0042] Penders, J. , Nagelkerke, A. , Cunnane, E. M. , Pedersen, S. V. , Pence, I. J. , Coombes, R. C. , & Stevens, M. M. (2021). Single particle automated raman trapping analysis of breast cancer cell‐derived extracellular vesicles as cancer biomarkers. ACS Nano, 15(11), 18192–18205. 34735133 10.1021/acsnano.1c07075 PMC9286313

[jev212419-bib-0043] Plottel, C. S. , & B. M. J. (2011). Microbiome and malignancy. Cell Host & Microbe, 10(4), 324–335. 22018233 10.1016/j.chom.2011.10.003 PMC3264051

[jev212419-bib-0044] Punnonen, K. , Hietanen, E. , Auvinen, O. , & Punnonen, R. (1989). Phospholipids and fatty acids in breast cancer tissue. Journal of Cancer Research and Clinical Oncology, 115(6), 575–578. 2606932 10.1007/BF00391361 PMC12211697

[jev212419-bib-0045] Riedl, S. , Rinner, B. , Asslaber, M. , Schaider, H. , Walzer, S. , Novak, A. , Lohner, K. , & Zweytick, D. (2011). In search of a novel target—phosphatidylserine exposed by non‐apoptotic tumor cells and metastases of malignancies with poor treatment efficacy. Biochimica Et Biophysica Acta, 1808(11), 2638–2645. 21810406 10.1016/j.bbamem.2011.07.026 PMC3175029

[jev212419-bib-0046] Rubenich, D. S. , Omizzollo, N. , Szczepański, M. J. , Reichert, T. E. , Whiteside, T. L. , Ludwig, N. , & Braganhol, E. (2021). Small extracellular vesicle‐mediated bidirectional crosstalk between neutrophils and tumor cells. Cytokine & Growth Factor Reviews, 61, 16–26. 34479816 10.1016/j.cytogfr.2021.08.002 PMC9235060

[jev212419-bib-0047] Skotland, T. , Ekroos, K. , Kauhanen, D. , Simolin, H. , Seierstad, T. , Berge, V. , Sandvig, K. , & Llorente, A. (2017). Molecular lipid species in urinary exosomes as potential prostate cancer biomarkers. European Journal of Cancer, 70, 122–132. 27914242 10.1016/j.ejca.2016.10.011

[jev212419-bib-0048] Smith, C. A. , Want, E. J. , O'Maille, G. , Abagyan, R. , & Siuzdak, G. X (2006). Processing mass spectrometry data for metabolite profiling using nonlinear peak alignment, matching and identification. Analytical Chemistry, 78, 779–787. 16448051 10.1021/ac051437y

[jev212419-bib-0049] St John, E. R. , Balog, J. , McKenzie, J. S. , Rossi, M. , Covington, A. , Muirhead, L. , Bodai, Z. , Rosini, F. , Speller, A. V. , Shousha, S. , & Ramakrishnan, R. (2017). Rapid evaporative ionisation mass spectrometry of electrosurgical vapours for the identification of breast pathology: Towards an intelligent knife for breast cancer surgery. Breast Cancer Research, 19(1), 1–14. 28535818 10.1186/s13058-017-0845-2 PMC5442854

[jev212419-bib-0050] Tay, T. K. Y. , & Tan, P. H. (2021). Liquid biopsy in breast cancer: A focused review. Archives of Pathology & Laboratory Medicine, 145(6), 678–686. 32045277 10.5858/arpa.2019-0559-RA

[jev212419-bib-0051] Tickner, J. A. , Urquhart, A. J. , Stephenson, S. A. , Richard, D. J. , & O'Byrne, K. J. (2014). Functions and therapeutic roles of exosomes in cancer. Frontiers in Oncology, 4, 127. 24904836 10.3389/fonc.2014.00127 PMC4034415

[jev212419-bib-0052] van Meer, G. , Voelker, D. R. , & Feigenson, G. W. (2008). Membrane lipids: Where they are and how they behave. Nature Reviews Molecular Cell Biology, 9(2), 112–124. 18216768 10.1038/nrm2330 PMC2642958

[jev212419-bib-0053] Venetis, K. , Cursano, G. , Pescia, C. , D'Ercole, M. , Porta, F. M. , Blanco, M. C. , Frascarelli, C. , Ivanova, M. , Rocco, E. G , & Fusco, N. (2023). Liquid biopsy: Cell‐free DNA based analysis in breast cancer. The Journal of Liquid Biopsy, 1, 100002. 10.1016/j.jlb.2023.100002 PMC11863823 40027284

[jev212419-bib-0054] Wymann, M. P. , & Schneiter, R. (2008). Lipid signalling in disease. Nature Reviews Molecular Cell Biology, 9(2), 162–176. 18216772 10.1038/nrm2335

[jev212419-bib-0055] Xavier, J. B. , Young, V. B. , Skufca, J. , Ginty, F. , Testerman, T. , Pearson, A. T. , Macklin, P. , Mitchell, A. , Shmulevich, I. , Xie, L. , & Caporaso, J. G. (2020). The cancer microbiome: Distinguishing direct and indirect effects requires a systemic view. Trends in Cancer, 6(3), 192–204. 32101723 10.1016/j.trecan.2020.01.004 PMC7098063

