# Supplementary material for: Extracellular vesicles as a promising source of lipid biomarkers for breast cancer detection in blood plasma
Source: J Extracell Vesicles. 2024 Mar 5;13(3):e12419. doi: 10.1002/jev2.12419 (PMC10914699; doi:10.1002/jev2.12419)
Supplement: Supplementary file 1 — Supporting Information [file JEV2-13-e12419-s001.docx]

**Extracellular vesicles as a promising source of lipid biomarkers for breast cancer detection in blood plasma**

Erika Dorado, M. Luisa Doria, Anika Nagelkerke, James S. McKenzie, Stefania Maneta-Stavrakaki, Thomas Whittaker, Jeremy Nicholson, R. Charles Coombes, Molly M Stevens, Zoltan Takats.

**Supplementary Table S1. List of lipids species enriched in breast cancer derived-EVs, when compared to their parental cells.** List of lipids significantly (*q*-value < 0.05) enriched (log_2_ fold change > 2) in breast cancer-derived EVs produced *in vitro* when compared to their parental cells (MCF-7, T47D, HCC1954, JIMT-1, MDA-MB-436, MDA-MB-231, MDA-MB-468, HCC1937 and Hs578T). Avg. rt = Average retention time.

| **m/z** | **Theoretical m/z** | **Avg. rt** | **Lipid ID** | **EVs/Cells** | **Formula** | **Ion** | **Log_2_ FC EVs/Cells** | **ANOVA q-value** |
| --- | --- | --- | --- | --- | --- | --- | --- | --- |
| 868.6721 | 868.6766 | 6.0 | PC(40:0) | EVs | C48H96NO8PNa | [M+Na]+ | 5.4 | 2.9E-06 |
| 687.5440 | 687.5447 | 7.4 | SM(d34:1) | EVs | C39H79N2O6P | [M-CH3]- | 3.9 | 3.4E-07 |
| 508.3395 | 508.3409 | 3.0 | LPE(20:0) | EVs | C25H52NO7P | [M-H]- | 3.4 | 2.6E-06 |
| 590.5514 | 590.5518 | 8.2 | Cer(d38:2) | EVs | C38H73NO3 | [M-H]- | 3.3 | 1.2E-04 |
| 659.5127 | 659.5134 | 6.7 | SM(d32:1) | EVs | C37H75N2O6P | [M-CH3]- | 3.3 | 1.3E-09 |
| 741.5908 | 741.5916 | 8.2 | SM(d38:2) | EVs | C43H85N2O6P | [M-CH3]- | 3.2 | 3.7E-06 |
| 840.6406 | 840.6453 | 5.3 | PC(38:0) | EVs | C46H92NO8PNa | [M+Na]+ | 3.2 | 9.7E-06 |
| 592.5693 | 592.5674 | 8.7 | Cer(d38:1) | EVs | C38H75NO3 | [M-H]- | 2.9 | 1.4E-05 |
| 510.4882 | 510.4892 | 6.9 | Cer(d32:0) | EVs | C32H65NO3 | [M-H]- | 2.8 | 3.6E-04 |
| 582.3770 | 582.3777 | 3.0 | LPC(18:0) | EVs | C26H54NO7P | [M+OAc]- | 2.8 | 1.8E-04 |
| 678.6767 | 678.6770 | 10.4 | Cer(d44:0) | EVs | C44H89NO3 | [M-H]- | 2.6 | 1.9E-03 |
| 820.6432 | 820.6437 | 9.0 | PE(O-38:0) | EVs | C43H88NO7P | [M+OAc]- | 2.6 | 1.1E-05 |
| 930.7168 | 930.7169 | 9.8 | PC(42:1) | EVs | C50H98NO8P | [M+OAc]- | 2.6 | 5.4E-05 |
| 622.6128 | 622.6144 | 9.5 | Cer(d40:0) | EVs | C40H81NO3 | [M-H]- | 2.6 | 5.7E-09 |
| 881.5175 | 881.5186 | 5.4 | PI(38:6) | EVs | C47H79O13P | [M-H]- | 2.5 | 1.6E-04 |
| 812.6105 | 812.6140 | 4.5 | PC(36:0) | EVs | C44H88NO8PNa | [M+Na]+ | 2.4 | 1.8E-06 |
| 618.5831 | 618.5831 | 8.9 | Cer(d40:2) | EVs | C40H77NO3 | [M-H]- | 2.3 | 2.5E-08 |
| 782.5727 | 782.5694 | 3.3 | PC(36:4) | EVs | C44H80NO8P | [M+H]+ | 2.3 | 1.7E-06 |
| 903.7483 | 903.7536 | 9.9 | SM(d44:0) | EVs | C49H101N2O6P | [M+OAc]- | 2.3 | 4.1E-02 |
| 620.5987 | 620.5987 | 9.3 | Cer(d40:1) | EVs | C40H79NO3 | [M-H]- | 2.2 | 9.8E-10 |
| 650.6454 | 650.6457 | 10.0 | Cer(d42:0) | EVs | C42H85NO3 | [M-H]- | 2.2 | 2.1E-10 |
| 564.5358 | 564.5361 | 8.1 | Cer(d36:1) | EVs | C36H71NO3 | [M-H]- | 2.2 | 5.4E-04 |
| 564.4027 | 564.4024 | 4.9 | LPE(24:1) | EVs | C29H58NO7P | [M+H]+ | 2.1 | 5.1E-04 |
| 562.5200 | 562.5205 | 7.5 | Cer(d36:2) | EVs | C36H69NO3 | [M-H]- | 2.1 | 3.8E-04 |
| 594.5830 | 594.5831 | 8.9 | Cer(d38:0) | EVs | C38H77NO3 | [M-H]- | 2.1 | 5.1E-04 |
| 508.4726 | 508.4735 | 6.7 | Cer(d32:1) | EVs | C32H63NO3 | [M-H]- | 2.0 | 7.1E-04 |
| 901.7356 | 901.7379 | 9.8 | SM(d44:1) | EVs | C49H99N2O6P | [M+OAc]- | 2.0 | 1.7E-02 |
| 756.6335 | 756.6359 | 8.4 | HexCer(d38:0) | EVs | C44H87NO8 | [M-H]- | 2.0 | 4.7E-03 |

**Supplementary Table S2. Lipids enriched in breast cancer cells, when compared to their secreted EVs.** List of lipids species enriched significantly (*q*-value < 0.05) enriched (log_2_ fold change > 2) in breast cancer cells (MCF-7, T47D, HCC1954, JIMT-1, MDA-MB-436, MDA-MB-231, MDA-MB-468, HCC1937 and Hs578T) when compared to their secreted EVs. Avg. rt = Average retention time.

| **m/z** | **Theoretical m/z** | **Avg. rt** | **Lipid ID** | **EVs/Cells** | **Formula** | **Ion** | **Log_2_ FC EVs/Cells** | **ANOVA *q*-value** |
| --- | --- | --- | --- | --- | --- | --- | --- | --- |
| 833.4652 | 833.4729 | 9.3 | PG(38:6) | Cells | C44H75O10PK | [M+K]+ | -7.2 | 1.8E-03 |
| 863.5121 | 863.5199 | 9.9 | PG(40:5) | Cells | C46H81O10PK | [M+K]+ | -6.1 | 2.6E-03 |
| 766.6920 | 766.6919 | 10.8 | TG(44:1) | Cells | C47H88O6 | [M+NH4]+ | -5.0 | 9.7E-03 |
| 956.8638 | 956.8641 | 11.9 | TG(58:4) | Cells | C61H110O6 | [M+NH4]+ | -4.9 | 4.6E-06 |
| 984.8931 | 984.8954 | 12.1 | TG(60:4) | Cells | C63H114O6 | [M+NH4]+ | -4.7 | 2.9E-06 |
| 822.7554 | 822.7545 | 11.4 | TG(48:1) | Cells | C51H96O6 | [M+NH4]+ | -4.6 | 8.6E-03 |
| 848.7705 | 848.7702 | 11.4 | TG(50:2) | Cells | C53H98O6 | [M+NH4]+ | -4.4 | 1.7E-04 |
| 874.7863 | 874.7858 | 11.5 | TG(52:3) | Cells | C55H100O6 | [M+NH4]+ | -4.3 | 9.0E-06 |
| 904.8335 | 904.8328 | 11.9 | TG(54:2) | Cells | C57H106O6 | [M+NH4]+ | -4.3 | 4.2E-05 |
| 876.8025 | 876.8015 | 11.7 | TG(52:2) | Cells | C55H102O6 | [M+NH4]+ | -4.2 | 8.3E-05 |
| 850.7869 | 850.7858 | 11.7 | TG(50:1) | Cells | C53H100O6 | [M+NH4]+ | -4.2 | 5.9E-03 |
| 978.8483 | 978.8484 | 11.6 | TG(60:7) | Cells | C63H108O6 | [M+NH4] | -4.1 | 5.3E-03 |
| 958.8782 | 958.8797 | 12.1 | TG(58:3) | Cells | C61H112O6 | [M+NH4]+ | -3.7 | 2.7E-05 |
| 986.9099 | 986.9110 | 12.3 | TG(60:3) | Cells | C63H116O6 | [M+NH4]+ | -3.6 | 9.4E-05 |
| 768.7080 | 768.7076 | 11.1 | TG(44:0) | Cells | C47H90O6 | [M+NH4]+ | -3.6 | 4.9E-02 |
| 980.8669 | 980.8641 | 11.8 | TG(60:6) | Cells | C63H110O6 | [M+NH4]+ | -3.5 | 2.6E-04 |
| 976.8326 | 976.8328 | 11.5 | TG(60:8) | Cells | C63H106O6 | [M+NH4]+ | -3.4 | 1.2E-04 |
| 926.8174 | 926.8171 | 11.6 | TG(56:5) | Cells | C59H104O6 | [M+NH4]+ | -3.4 | 6.3E-06 |
| 950.8172 | 950.8171 | 11.4 | TG(58:7) | Cells | C61H104O6 | [M+NH4]+ | -3.3 | 1.2E-04 |
| 868.7326 | 868.7389 | 11.3 | TG(52:6) | Cells | C55H94O6 | [M+NH4]+ | -3.1 | 5.2E-04 |
| 952.8334 | 952.8328 | 11.6 | TG(58:6) | Cells | C61H106O6 | [M+NH4]+ | -3.1 | 3.5E-06 |
| 307.2632 | 307.2643 | 3.8 | FA(20:2) | Cells | C20H36O2 | [M-H]- | -3.1 | 1.9E-06 |
| 303.2320 | 303.2330 | 3.0 | FA(20:4) | Cells | C20H32O2 | [M-H]- | -3.0 | 1.0E-05 |
| 911.8084 | 911.8062 | 11.7 | TG(56:4) | Cells | C59H106O6 | [M+H]+ | -3.0 | 1.6E-04 |
| 329.2475 | 329.2486 | 3.1 | FA(22:5) | Cells | C22H34O2 | [M-H]- | -2.8 | 2.2E-09 |
| 305.2476 | 305.2486 | 3.3 | FA(20:3) | Cells | C20H34O2 | [M-H]- | -2.8 | 4.7E-11 |
| 822.5353 | 822.5291 | 7.4 | PE(38:6) | Cells | C43H74NO8P | [M+OAc]- | -2.6 | 3.5E-04 |
| 924.8001 | 924.8015 | 11.3 | TG(56:6) | Cells | C59H102O6 | [M+NH4]+ | -2.5 | 5.7E-06 |
| 810.5260 | 810.5291 | 7.7 | PS(38:4) | Cells | C44H78NO10P | [M-H]- | -2.3 | 5.1E-07 |
| 680.4481 | 680.4497 | 3.4 | PS(28:0) | Cells | C34H66NO10P | [M+H]+ | -2.2 | 9.3E-03 |
| 736.5168 | 736.5124 | 6.8 | HexCer(d34:2) | Cells | C40H75NO8K | [M+K]+ | -2.1 | 5.0E-02 |
| 770.5740 | 770.5670 | 6.9 | PE(36:0) | Cells | C41H82NO8PNa | [M+Na]+ | -2.1 | 2.6E-05 |
| 824.5421 | 824.5447 | 7.7 | PE(38:5) | Cells | C43H76NO8P | [M+OAc]- | -2.1 | 6.8E-07 |
| 741.4694 | 741.4712 | 7.4 | PG(34:4) | Cells | C40H71O10P | [M-H]- | -2.1 | 9.2E-08 |
| 788.5425 | 788.5447 | 6.2 | PS(36:1) | Cells | C42H80NO10P | [M-H]- | -2.1 | 7.0E-06 |

**Supplementary Table S3. Lipids with very strong correlation between EVs and their parental cells.** List of lipids species with a Spearman rank-order correlation coefficient r > 0.8 indicating very strong correlation between EVs and their parental cells, with a *p*-value < 0.05 indicating that the correlation is statistically significant. Avg. rt = Average retention time.

| **m/z** | **Theoretical m/z** | **Avg. rt** | **Lipid ID** | **Formula** | **Ion** | **Spearman rs** | **Spearman *p*-value** |
| --- | --- | --- | --- | --- | --- | --- | --- |
| 886.6993 | 886.6907 | 9.27 | PC(O-40:2) | C48H94NO7P | [M+OAc]- | 0.96 | 1.26E-15 |
| 808.5468 | 808.5498 | 8.05 | PE(O-38:6) | C43H76NO7P | [M+OAc]- | 0.93 | 1.24E-12 |
| 780.6357 | 780.6359 | 8.29 | HexCer(d40:2) | C46H87NO8 | [M-H]- | 0.92 | 5.92E-12 |
| 782.6502 | 782.6515 | 8.88 | HexCer(d40:1) | C46H89NO8 | [M-H]- | 0.91 | 7.43E-11 |
| 810.6826 | 810.6828 | 9.44 | HexCer(d42:1) | C48H93NO8 | [M-H]- | 0.91 | 8.68E-11 |
| 672.4971 | 672.4974 | 7.33 | PE(O-32:2) | C37H72NO7P | [M-H]- | 0.90 | 1.58E-10 |
| 698.5570 | 698.5576 | 6.85 | HexCer(d34:1) | C40H77NO8 | [M+OAc]- | 0.89 | 5.89E-10 |
| 884.6827 | 884.6750 | 8.68 | PC(O-40:3) | C48H92NO7P | [M+OAc]- | 0.89 | 5.89E-10 |
| 844.6862 | 844.6437 | 9.06 | PE(O-40:2) | C45H88NO7P | [M+OAc]- | 0.88 | 1.03E-09 |
| 674.5128 | 674.5130 | 6.98 | PC(O-30:1) | C38H76NO7P | [M-CH3]- | 0.88 | 1.42E-09 |
| 646.4813 | 646.4817 | 7.17 | PE(O-30:1) | C35H70NO7P | [M-H]- | 0.85 | 1.40E-08 |
| 772.5284 | 772.5287 | 7.28 | PE(O-40:8) | C45H76NO7P | [M-H]- | 0.85 | 2.14E-08 |
| 760.5294 | 760.5498 | 7.54 | PE(O-34:2) | C39H76NO7P | [M+OAc]- | 0.85 | 2.46E-08 |
| 722.4971 | 722.4978 | 5.81 | PE(30:0) | C35H70NO8P | [M+OAc]- | 0.83 | 8.08E-08 |
| 854.6726 | 854.6644 | 8.86 | PE(44:2) | C49H94NO8P | [M+OAc]- | 0.83 | 8.42E-08 |
| 662.4761 | 662.4766 | 5.81 | PE(30:0) | C35H70NO8P | [M-H]- | 0.83 | 1.12E-07 |
| 780.5906 | 780.5913 | 8.89 | PE(O-40:4) | C45H84NO7P | [M-H]- | 0.82 | 1.22E-07 |
| 750.5439 | 750.5443 | 7.85 | PC(O-36:5) | C44H80NO7P | [M-CH3]- | 0.82 | 1.27E-07 |
| 645.4531 | 645.4501 | 6.72 | PA(32:1) | C35H67O8P | [M-H]- | 0.82 | 1.37E-07 |
| 790.5960 | 790.5968 | 7.84 | PE(O-36:1) | C41H82NO7P | [M+OAc]- | 0.82 | 1.60E-07 |
| 920.6306 | 920.6386 | 6.61 | PC(42:6) | C50H88NO8P | [M+OAc]- | 0.82 | 1.80E-07 |
| 856.6895 | 856.6801 | 9.16 | PE(44:1) | C49H96NO8P | [M-H]- | 0.82 | 1.80E-07 |
| 706.5701 | 706.5745 | 7.61 | PE(O-34:0) | C39H80NO7P | [M+H]+ | 0.82 | 2.10E-07 |
| 864.6811 | 864.6477 | 9.1 | PC(42:5) | C50H90NO8P | [M+H]+ | 0.81 | 2.63E-07 |
| 696.4970 | 696.4974 | 7.24 | PE(O-34:4) | C39H72NO7P | [M-H]- | 0.81 | 3.16E-07 |
| 750.5640 | 750.5655 | 7.02 | PC(O-30:0) | C38H78NO7P | [M+OAc]- | 0.81 | 3.93E-07 |
| 756.5845 | 756.5902 | 8.79 | PE(O-38:3) | C43H82NO7P | [M+H]+ | 0.81 | 4.07E-07 |
| 804.6115 | 804.6124 | 7.84 | PC(O-34:1) | C42H84NO7P | [M+OAc]- | 0.80 | 4.69E-07 |

**Supplementary Table S4. Nanoparticle tracking analysis (NTA) of the EVs isolated from blood plasma.** EVs’ concentration and size (mean and mode) for the EVs isolated from blood plasma from healthy volunteers (HV), patients with primary breast cancer (Primary) and patients with progressive metastatic breast cancer (Metastatic). N=5 60 s videos per sample captured on the NanoSight.

| **Sample** | **Concentration (particles/mL)** | **± SD** | **Mean particle size (nm)** | **Mode particle size (nm)** | **± SD** |
| --- | --- | --- | --- | --- | --- |
| HV | 2.07E+09 | 1.16E+08 | 165.8 | 135.2 | 65.2 |
| HV | 1.38E+09 | 3.39E+08 | 159.9 | 145.7 | 49.9 |
| HV | 1.73E+09 | 2.31E+08 | 185.7 | 148.6 | 62.4 |
| HV | 2.15E+09 | 1.32E+08 | 171.9 | 142.7 | 68.6 |
| HV | 3.24E+09 | 2.78E+08 | 140.6 | 98.1 | 57.8 |
| HV | 1.15E+09 | 1.4E+08 | 189.3 | 183.2 | 55.4 |
| HV | 1.96E+09 | 1.32E+08 | 169.3 | 128.3 | 63.6 |
| HV | 1.67E+09 | 1.28E+08 | 169.5 | 134.9 | 51.5 |
| HV | 2.41E+09 | 1.46E+08 | 157.6 | 130.8 | 56.9 |
| HV | 3E+09 | 1.4E+08 | 166.3 | 143 | 53.8 |
| Primary | 1.08E+10 | 1.16E+08 | 162.7 | 126.3 | 56.6 |
| Primary | 7.25E+09 | 4.52E+08 | 152.8 | 105.7 | 57.6 |
| Primary | 1.36E+10 | 1.64E+08 | 183.9 | 154.7 | 61.8 |
| Primary | 1.26E+10 | 2.26E+09 | 176.2 | 125.5 | 72.2 |
| Primary | 2.23E+09 | 1.33E+08 | 185.5 | 140.7 | 73.4 |
| Primary | 1.22E+10 | 1.65E+08 | 162.5 | 119.6 | 58.3 |
| Primary | 5.6E+09 | 1.75E+08 | 161.6 | 129.2 | 67.7 |
| Primary | 3.55E+09 | 2.08E+08 | 150.9 | 110.4 | 53.8 |
| Primary | 9.25E+09 | 5.87E+08 | 167.1 | 126.2 | 63.5 |
| Primary | 7.59E+09 | 2.76E+08 | 163.6 | 123.2 | 59.7 |
| Metastatic | 1.8E+09 | 1.64E+08 | 227.5 | 179.5 | 79.7 |
| Metastatic | 4.15E+09 | 2.9E+08 | 175.5 | 134.8 | 78.3 |
| Metastatic | 4.18E+09 | 1.84E+08 | 151.5 | 119.5 | 63.4 |
| Metastatic | 3.4E+09 | 1.7E+08 | 163.1 | 134.9 | 69.7 |
| Metastatic | 7.02E+09 | 5.98E+08 | 139.4 | 104.5 | 59.5 |
| Metastatic | 4.94E+09 | 4.56E+08 | 149.1 | 106.2 | 67.3 |
| Metastatic | 3.43E+09 | 1.94E+08 | 164.4 | 119.9 | 62.7 |
| Metastatic | 2.21E+09 | 2.72E+08 | 188.9 | 169.7 | 61.2 |
| Metastatic | 3.18E+09 | 2.28E+08 | 211.3 | 182.7 | 69.3 |


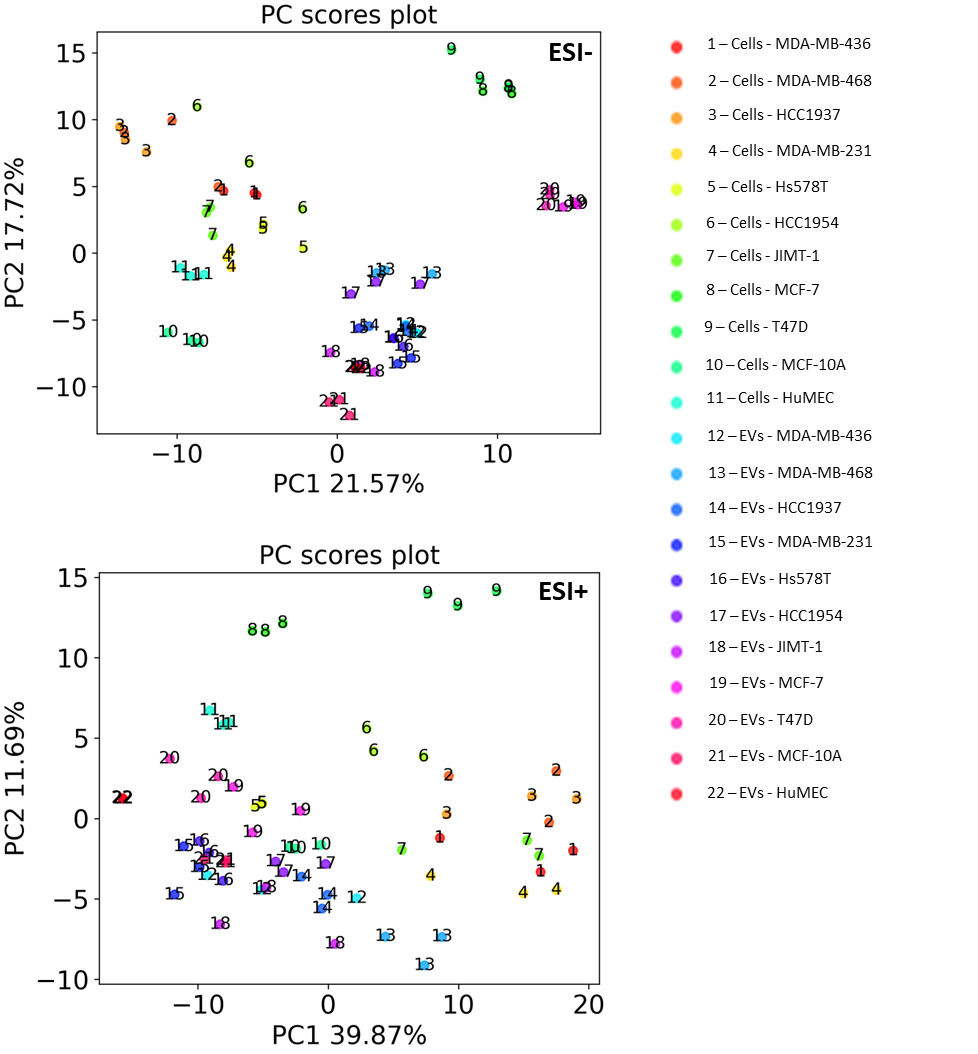


**Supplementary Figure S1. Lipidomic analysis of EVs and their parental cells.** PCA showing the LC-MS data distribution for both cells (N=11 cell lines and 3 biological replicates; n=33) and their secreted EVs (n=33). This includes both cancerous and non-cancerous cells and their EVs. The LC-MS data was acquired in both ESI+ and ESI- modes.


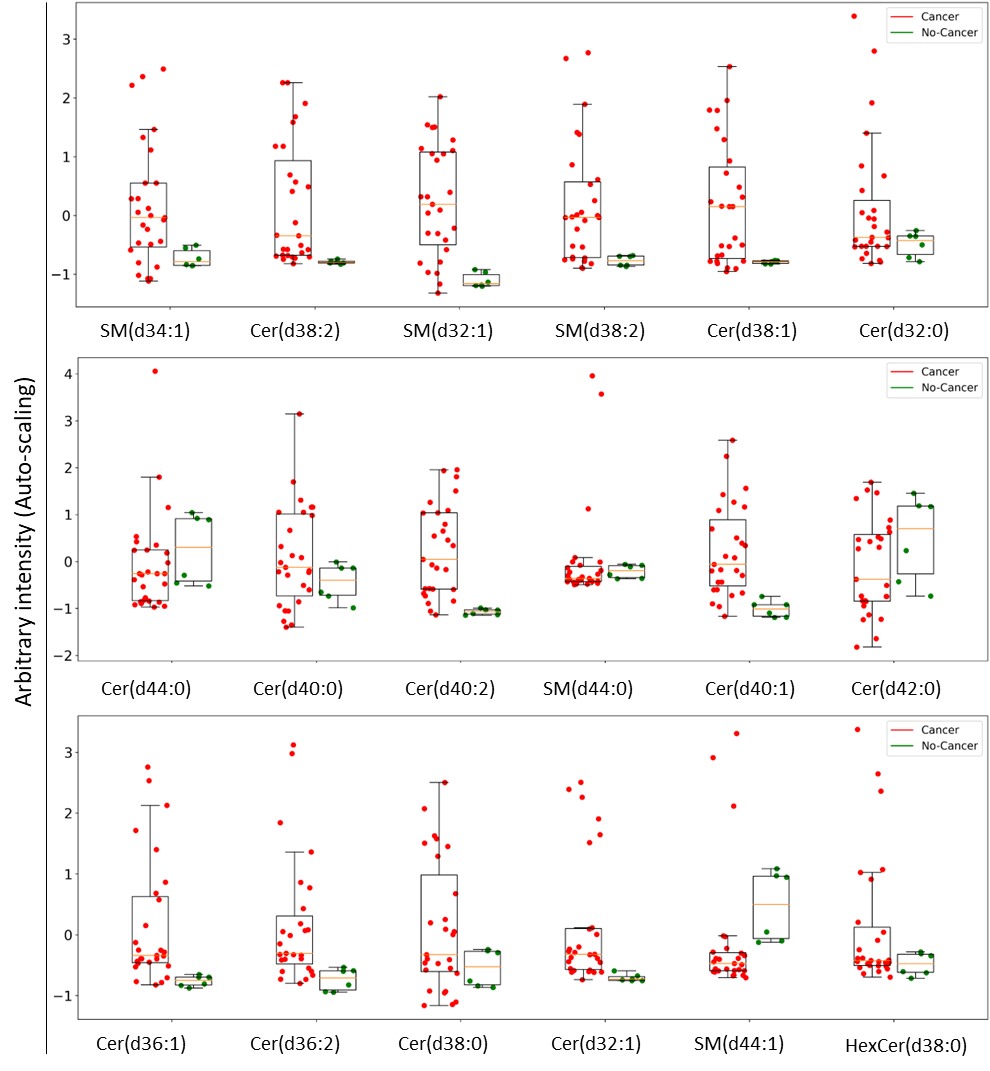


**Supplementary Figure S2. Boxplots of cancer and no-cancer derived EVs based on sphingolipids enriched in breast cancer derived-EVs when compared to their parental cells.** EVs produced by cancerous (N=9 breast cancer cell lines, 3 biological replicates; n=27) and non-cancerous (N=2 non-cancerous cell lines and their respective EVs, 3 biological replicates; n=6) cells.


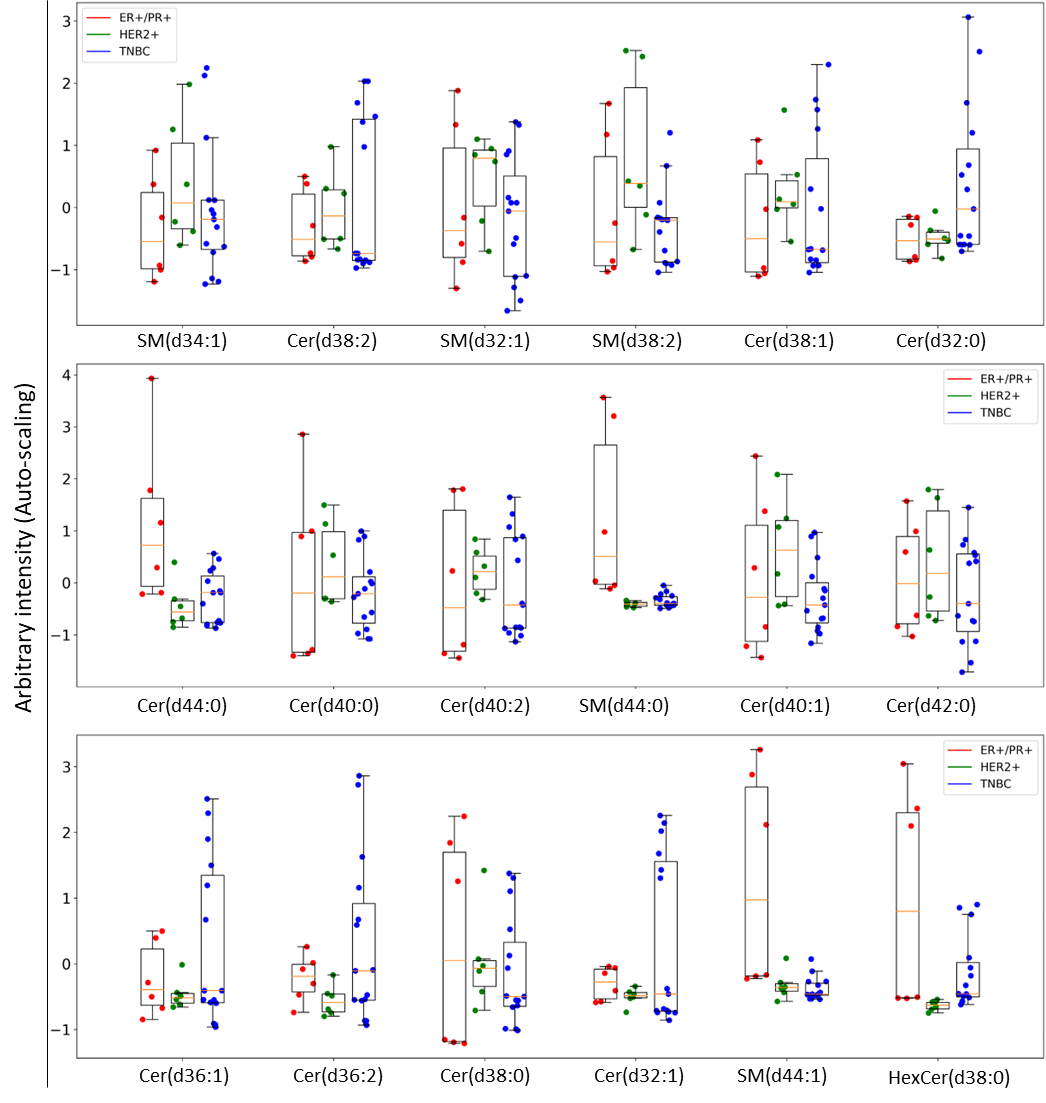


**Supplementary Figure S3. Box plots for breast cancer derived-EVs classified into their respective breast cancer subtypes based on sphingolipids enriched in breast cancer derived-EVs when compared to their parental cells.** EVs produced by breast cancer cell lines from different molecular subtypes: ER+/PR+ (N=2 cell lines and 3 biological replicates; n=6), HER2+ (N=2 cell lines, 3 biological replicates; n=6), and TNBC (N=5 cell lines, 3 biological replicates; n=15).
